# Whole Grains, Refined Grains, and Cancer Risk: A Systematic Review of Meta-Analyses of Observational Studies

**DOI:** 10.3390/nu12123756

**Published:** 2020-12-07

**Authors:** Glenn A. Gaesser

**Affiliations:** College of Health Solutions, Arizona State University, Phoenix, AZ 85004, USA; glenn.gaesser@asu.edu; Tel.: +1-602-827-2283

**Keywords:** diet, fiber, cereals, chronic disease, epidemiology, cohort, case-control, mortality

## Abstract

PubMed, Web of Science, and the Cochrane Database of Systematic Reviews were searched for meta-analyses that provided risk estimates (±95% confidence intervals) for associations between intakes of whole and refined grains and risk of total and site-specific cancer. The preferred reporting items for systematic reviews and meta-analyses (PRISMA) guidelines were followed. Only meta-analyses that included whole grains and refined grains as separate food groups, and not as part of dietary patterns, were included. A total of 17 publications were identified that met inclusion criteria. Within these, results from a total of 54 distinct meta-analyses were reported for whole grains and 5 meta-analyses for refined grains. For total cancer mortality, 7 meta-analyses of cohort studies indicated that whole grain intake was associated with 6% to 12% lower risk in comparison of highest vs. lowest intake groups, and 3% to 20% lower risk for doses ranging from 15 to 90 g/day. For site-specific cancers, meta-analyses indicated that whole grain intake was consistently associated with lower risks of colorectal, colon, gastric, pancreatic, and esophageal cancers. Limited data were available for refined grains, with only 4 publications providing risk estimates, and only 1 of the meta-analyses included more than 3 studies. High intake of refined grains was associated with increased risk of colon and gastric cancer. By contrast, in the only dose-response meta-analysis, each 90 g/day consumption of refined grains was associated with a 6% lower risk of total cancer. In addition to the limited number of published meta-analyses on refined grains, results were also weakened due to the fact that refined grains were frequently defined to include both staple grain foods and indulgent grain foods, and the majority of studies included in the meta-analyses provided no specific definition of refined grains. Overall, meta-analyses of cohort and case-control studies consistently demonstrate that whole grain intake is associated with lower risk of total and site-specific cancer, and support current dietary recommendations to increase whole grain consumption. By contrast, the relationship between refined grain intake and cancer risk is inconclusive.

## 1. Introduction

Whole grains are associated with reduced risk of a number of chronic diseases, including cancer, and are recommended as an important part of a healthy diet [1,2,3]. By contrast, refined grains do not have similarly beneficial inverse associations between consumption and disease risk [4,5,6]. Consequently, dietary guidelines encourage increased consumption of whole grains and reduced consumption of refined grains [3]. The American Association of Cereal Chemists defined whole grains as consisting of the “intact, ground, cracked or flaked caryopsis, whose principal anatomical components—the starchy endosperm, germ and bran—are present in the same relative proportions as they exist in the intact caryopsis” [7]. This definition was adopted by regulatory and health promotion organizations to encourage greater consumption of whole grain foods [8]. Refined grains have some or all of the bran layers removed during processing, which reduces the content of fiber and micronutrients [8]. In research studies, whole grain and refined grain intake is assessed in terms of consumption of grain-based foods which may contain varying percentages of whole grains and refined grains, and definitions vary among studies [5,8]. In addition, grains may be consumed as a single food or as an ingredient in foods [9].

Much of the published research used to inform dietary guidelines comes from cohort studies that have examined the association between dietary patterns and risk of various chronic diseases [10]. A healthy dietary pattern typically includes whole grains, fruits, vegetables, fish, legumes, nuts, and low-fat dairy products. In contrast, an unhealthy dietary pattern, also widely referred to as a Western dietary pattern, is characterized by consumption of red and processed meat, sugar-sweetened foods and beverages, French fries, high-fat dairy products, and refined grains. With regard to cancer, the healthy dietary pattern is frequently associated with reduced risk and the Western dietary pattern is usually associated with increased risk [11,12,13,14,15]. However, dietary patterns do not allow for the contribution to cancer risk of specific food groups, such as whole and refined grain foods. Limitations of dietary pattern research, particularly with respect to interpretation of health outcomes associated with refined grain intake, has been reviewed recently [5]. For example, within the Western dietary pattern, consumption of red and processed meat is associated with increased risk of colorectal cancer and all-cause mortality, but consumption of refined grains is not [16,17].

Therefore, the objective of this review was to summarize the published research on the association between whole grain intake and cancer risk and between refined grain intake and cancer risk (including incidence and mortality). The review focuses exclusively on results from meta-analyses of observational cohort and case-control studies of adults in which whole grains and refined grains were evaluated as distinct food categories and not as part of dietary patterns.

## 2. Materials and Methods

The preferred reporting items for systematic reviews and meta-analyses (PRISMA) guidelines were used for this review [18]. The Institute for Scientific Information’s Web of Science, PubMed, and the Cochrane Database of Systematic Reviews were used to identify relevant meta-analyses, and were searched from database inception until September 1, 2020. The search strategy included the following terms: “Whole grain” OR “wholegrain” OR “whole-grain” OR “whole grains” OR “wholegrains” OR “whole-grains” OR “refined grain” OR “refined grains” AND “cancer”. Search results were further filtered by “meta-analysis”, “systematic review”, or “review”. No restrictions were placed on date of publication. Only English language publications were considered. Reference lists and electronic citation records of all identified meta-analyses were also reviewed for additional meta-analyses not found in the initial searches. Meta-analyses of cohort and/or case-control studies were included in this review if they provided relative risks or odds ratios with 95% confidence intervals, for total or site-specific cancer risk associated with either whole grain or refined grain intake. Whole grain and refined grain intake had to be considered as separate food groups, and not as part of a dietary pattern. Meta-analyses that included whole grains or refined grains as part of dietary patterns were excluded.

**Figure 1:**
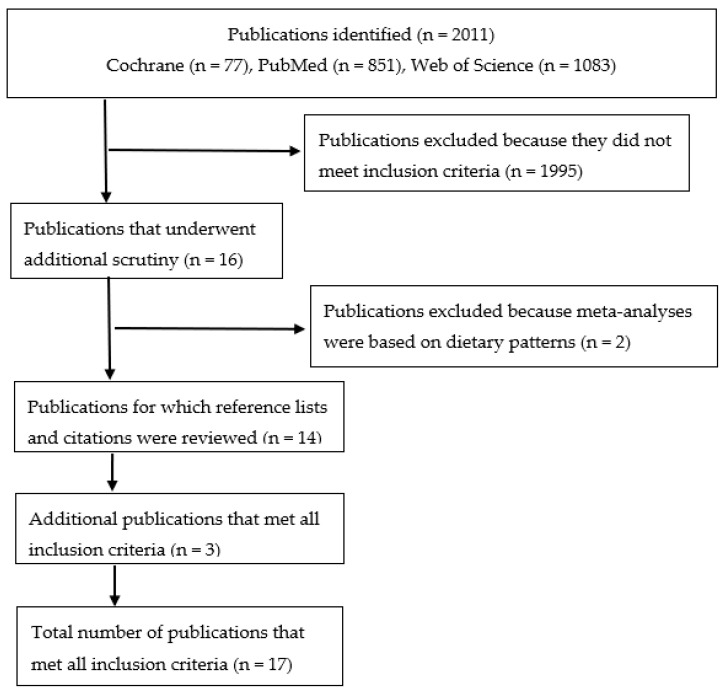
Flow chart of publication selection (see text for further details on search terms and filters used).

The flow chart for publication selection is presented in Figure 1. The initial searches identified 77 (Cochrane), 851 (PubMed), and 1083 (Web of Science) articles. After further restricting those searches using the filters described above, 16 publications were found that underwent additional scrutiny to ensure that they met the inclusion criteria [16,19,20,21,22,23,24,25,26,27,28,29,30,31,32,33]. Two of the publications were excluded because some or all of the studies used in those meta-analyses included whole grains as part of a dietary pattern [23,27]. Review of reference lists and electronic citation records of each of the meta-analyses identified in the search revealed three additional publications that met inclusion criteria [34,35,36]. Thus, a total of 17 publications, each with 1 or more meta-analyses included in the publication, were used in the current review.

## 3. Results

Details of the meta-analyses, including information on the number of cohort and case-control studies included, total number of participants, and results of publication bias and heterogeneity assessments, are presented in Supplementary Table S1. Details on each of the studies used in the meta-analyses for whole grains and refined grains, including cohort and case-control populations, method of dietary assessment, and definition of whole grains and refined grains, are presented in Supplementary Tables S2 and S3, respectively.

All cohort studies were from the United States and Europe (United Kingdom, Norway, Sweden, Denmark, Finland, Spain) (Supplemental Tables S2 and S3). Cohorts included men and women aged 30–87 years at baseline, and ranged in size from 7216 to 489,611 participants. The case-control studies mainly included populations from the United States and Europe (Italy, Germany, Poland, Sweden, The Netherlands, Belgium, Switzerland, Greece, Spain), but also from Asia (China, Japan, South Korea, India), the Middle East (Jordan, Iran), South America (Brazil, Uruguay), Canada, and Australia (Supplemental Tables S2 and S3). Case-control studies included fewer participants, with most studies including fewer than 1000 cases and controls. Publication bias was assessed in all but two of the meta-analyses, mostly by using Egger’s [37] or Begg’s [38] tests, while one used the risk of bias in systematic reviews ROBIS assessment tool [39]. In all but one meta-analysis [32], no significant bias was detected (Supplementary Table S1). Heterogeneity was assessed with the I^2^ statistic [40]. Twenty-one of the meta-analyses reported statistically significant heterogeneity in their meta-analyses, with I^2^ between 53% and 91% (Supplementary Table S1).

### 3.1. Whole Grain Intake and Total Cancer Risk

Eight meta-analyses have been published on the relationship between whole grain intake and either cancer mortality [20,21,22,26,29,31,33] or total cancer risk [24] (Table 1). Seven of the meta-analyses used data entirely [20,21,22,26,29,33] or predominantly [31] from cohort studies, and reported relative risks for total cancer mortality. One used only case-control studies [24], and reported on total cancer risk from multiple sites combined. All 8 of the meta-analyses provided risk estimates for categorial analyses (highest vs. lowest intakes), and 7 reported risk estimates for dose-response analyses. In virtually all instances, whole grain intake was associated with significantly lower cancer risk in both highest intake vs. lowest intake analyses and in dose-response analyses (Table 1). For the meta-analyses of cohort studies, high intake of whole grains was associated with a 6% to 12% lower risk of cancer mortality [20,21,22,26,29,31,33]. In the dose-response analyses, doses between 50 and 90 g/day were associated with a 9% to 20% lower cancer mortality risk [20,21,22,29,33]. Doses in the range of 15 to 30 g/day were associated with risk reductions of 3% to 11% [26,31,33]. Collectively, the 7 dose-response analyses presented in Table 1 indicate that each 30 g/day intake of whole grains (~1 serving) is associated with a ~7% reduction in cancer mortality risk.

In the one meta-analysis that relied solely on case-control studies [24], a pooled analysis of 40 case-control studies (Table 1) indicated that when comparing the highest vs. lowest intake of whole grains, the highest intake category was associated with a 34% lower risk of cancer. 

In summary, even though 8 meta-analyses have been published on the association between whole grain intake and total cancer mortality, it must be noted that 6 of meta-analyses [20,21,22,26,29,33] relied on essentially the same 6 cohort studies for determination of risk estimates [81,82,83,84,85,87], all from the United States or Europe. The data from Huang et al. (NIH-AARP Diet and Health Study) [82], Jacobs et al. (Iowa Women’s Health Study) [83], and Wu et al. (Nurses’ Health Study I and Health Professionals Follow-up Study) [85] were used in all 6 of these meta-analyses, the data from Buil–Cosiales et al. (PREDIMED trial) [81] and Johnsen et al. (HELGA cohort) [84] were used in 5 of the meta-analyses, and data from Jacobs et al. (Norwegian County Study) [87] were used in 4 of these meta-analyses (see Supplementary Table S2). Thus, it is not surprising that the risk reductions associated with whole grain intake were similar across these meta-analyses.

### 3.2. Whole Grain Intake and Site-Specific Cancer Risk

Eleven of the published meta-analyses provided risk estimates for whole grain intake and site-specific cancer risk, including colorectal [16,19,24,26,28,32], colon and rectal [16,19,28], gastric [24,32,35,36], pancreatic [24,25], breast [24,30], prostate [26,34], esophageal [24,32], oral [24], endometrial [24], brain [24], and non-Hodgkin’s lymphoma [24]. All results are presented in Table 2 and are described below. With few exceptions, whole grain intake was associated with lower risk of site-specific cancers. 

#### 3.2.1. Colorectal, Colon, and Rectal Cancer

Without exception, all six meta-analyses reported significantly lower colorectal cancer risk associated with whole grain intake (Table 2). Four of these were based entirely on cohort studies [16,19,26,28], one entirely on case-control studies [24], and one used both cohort and case-control studies [32]. In the five meta-analyses comparing the highest vs. lowest intake groups, participants with the highest intake of whole grains had an 11% to 21% lower risk of colorectal cancer. In the four dose-response analyses, colorectal cancer risk was reduced by 17% for each 90 g/day [19,28], by 5% for each 30 g/day [16], and by 3% for each 15 g/day [26]. When adjusting for dose, the combined results of these four dose-response analyses [16,19,26,28] suggest that colorectal cancer risk is reduced by approximately 15% to 17% per 90 g/day intake of whole grains.

For colon cancer, all three meta-analyses reported lower risk associated with whole grain intake. In the highest vs. lowest intake comparisons, high intake of whole grains was associated with a 15% to 18% lower risk of colon cancer. In the dose-response analyses, colon cancer risk was reduced by 14% to 18% for each 90 g/day [19,28] and by 3% for each 30 g/day [16] consumption of whole grains.

By contrast, for rectal cancer only one of the three meta-analyses reported a significantly lower risk associated with whole grain intake. Although the relative risks in categorical and dose-response analyses ranged between 0.80 and 0.94, only the 20% lower risk reported by Schwingshackl et al. [16] in the highest vs. lowest intake comparisons was statistically significant.

#### 3.2.2. Gastric Cancer

Four meta-analyses have been published on the association between whole grain intake and gastric cancer risk [24,32,35,36]. All four, which relied primarily [32,35,36] or entirely [24] on case-control studies, reported 13% to 39% lower risk when comparing highest vs. lowest intake groups. No dose-response analyses have been published.

#### 3.2.3. Pancreatic Cancer

Two meta-analyses have been published on the association between whole grain intake and pancreatic cancer risk. In a meta-analysis of 4 case-control studies, Jacobs et al. [24] reported a 30% lower risk of pancreatic cancer in a comparison of highest vs. lowest consumption of whole grains. Using data from 3 case-control studies, one cohort study, and the point estimate from the earlier meta-analysis by Jacobs et al. [24], the meta-analysis by Lei et al. [25] indicated a 24% lower risk of pancreatic cancer in comparing highest vs. lowest intakes of whole grains.

#### 3.2.4. Prostate Cancer

Three meta-analyses have been published on the association between whole grain intake and risk of prostate cancer, including 2 categorical analyses [26,34] and 1 dose-response analysis [26]. In contrast to the site-specific cancers discussed above, none of the meta-analyses indicated a benefit for whole grain intake. In fact, one of the meta-analyses indicated a 10% higher risk of prostate cancer when comparing highest vs. lowest intake groups [26] (Table 2).

#### 3.2.5. Breast Cancer

Three meta-analyses have been published on breast cancer, including 2 categorical analyses [24,30] and 1 dose-response analysis [30]. In the meta-analysis by Xiao et al. [30], which included 4 cohort studies and 7 case-control studies, whole grain intake was associated with a 16% lower risk of breast cancer when comparing highest vs. lowest intake categories. In the dose-response analysis that included 3 cohort studies and 3 case-control studies, each 50 g per day increase in whole grain intake was associated with a 17% lower risk of breast cancer. 

In an earlier meta-analysis by Jacobs et al. [24], which included only 2 case-control studies, whole grain consumption was not associated with a significantly lower risk of breast cancer, although the odds ratio of 0.86 was similar to the statistically significant relative risk of 0.84 reported in the more recent meta-analysis of Xiao et al. [30] (Table 2).

#### 3.2.6. Esophageal Cancer

Both meta-analyses on esophageal cancer, using mostly [32] or entirely [24] case-control studies, reported 46% to 48% lower risk of esophageal cancer when comparing the highest vs. lowest intakes of whole grain.

#### 3.2.7. Other Cancers

Limited data have been published on other cancers, including oral, brain, endometrial, and non-Hodgkin’s lymphoma. For each of these cancers, only one meta-analysis has been published, using case-control studies [24]. In categorical analyses, the highest intake group for whole grains was associated with a 43% lower risk of oral/pharyngeal/tongue cancer, a 33% lower risk of brain cancer, a 45% lower risk of endometrial cancer, and a 59% lower risk of non-Hodgkin’s lymphoma.

### 3.3. Refined Grain Intake and Cancer

In contrast to whole grains, only a few meta-analyses have been published on the association between refined grain intake and cancer, including total cancer [20], colorectal cancer [16], colon cancer [16], and gastric cancer [35,36]. All results are presented in Table 3. 

#### 3.3.1. Total Cancer

In a dose-response meta-analysis by Aune et al. [20], which included 2 cohort studies [83,85], each 90 g per day consumption of refined grains was associated with a 6% lower risk of total cancer. 

#### 3.3.2. Colorectal Cancer

Schwingshackl et al. [16] included 3 cohort studies in meta-analyses on the association between refined grain intake and colorectal cancer [95,103] and colon cancer [95,112]. In comparison of the highest vs. lowest intakes, refined grain consumption was not associated with colorectal cancer but was associated with a 27% higher risk of colon cancer.

#### 3.3.3. Gastric Cancer

Two meta-analyses have reported on the association between refined grain intake and risk of gastric cancer. Using highest vs. lowest intake comparisons from 18 case-control studies, Wang et al. [35] reported that refined grain intake was associated with a 36% higher risk of gastric cancer. In a smaller meta-analysis by Xu et al. [36], which included 1 cohort study and 2 case-control studies, the highest intake group of refined grains had a 65% greater risk of gastric cancer compared to the lowest intake group.

## 4. Discussion

Published meta-analyses reviewed herein demonstrate that whole grain intake is consistently associated with lower risk of total cancer mortality. This was observed in both categorical and dose-response analyses. Consistently lower risks for colorectal, colon, gastric, pancreatic, and esophageal cancers were also observed. These findings are congruent with the findings of expert reports and public health recommendations that advocate for greater consumption of whole grains [2,3,10]. Many countries include whole grains in their dietary recommendations [153]. For example, the United States dietary guidelines recommend that Americans consume at least 3 servings per day of whole grains, with a serving defined as 1 ounce-equivalent (~28 g) of a whole grain food [3]. Only 2% to 7% of the U.S. population achieves this goal [154,155], with whole grain intake the averaging <1 serving per day [10,155]. Consequently, the dose-response analyses in Table 1 and Table 2 suggest that significant reductions in total, colorectal, and colon cancer risk may be achieved by increasing whole grain intake above current levels.

In contrast to these findings, whole grain intake was not associated with decreased risk of prostate cancer. Only two publications reported meta-analyses on whole grain intake and prostate cancer risk (Table 2). The only meta-analysis that reported a higher prostate cancer risk was associated with whole grain intake included only 3 studies. It is also important to note that of the 9 studies included in the meta-analyses on prostate cancer, 4 of them did not provide a definition of whole grains (Supplementary Table S2). Thus, conclusions about the association between whole grain intake and prostate cancer should be interpreted with these limitations in mind.

Unlike the results for whole grains, meta-analyses revealed no consistent findings on the association between refined grain intake and cancer risk. Three of the meta-analyses indicated that refined grain intake may be associated with higher risk of colon and gastric cancer. In contrast, one meta-analysis reported a 6% lower risk of total cancer mortality associated with higher intake of refined grains. There are several important limitations to the meta-analyses on refined grains, and these are discussed below. Nevertheless, the limited and inconsistent findings from these meta-analyses on refined grains analyzed as a separate food category are not entirely supportive of the data from dietary pattern analyses showing that a Western dietary pattern that includes refined grains is usually associated with higher risk of cancer [11,12,13,14,15].

### 4.1. Mechanisms for Reduced Cancer Risk Associated with Whole Grain Intake

Whole grains may reduce cancer risk via a number of mechanisms, as reviewed previously [9,156,157,158]. Whole grain intake is correlated with cereal fiber intake [159,160,161]. Three meta-analyses have shown that cereal fiber is associated with reduced cancer risk [19,162,163], and two of these focused solely on colorectal cancer risk [19,162]. This may be particularly relevant for interpretation of the consistent findings of meta-analyses showing whole grain intake associated with reduced risk of colorectal cancer [16,19,24,26,28,32]. Cereal fiber increases fecal bulk and reduces gastrointestinal transit time [164], which could dilute carcinogens and reduce their absorption. Whole grain consumption, particularly from wheat, increases production of short-chain fatty acids, such as butyrate. Butyrate is a major energy source of normal human colon cells [165]. Butyrate has also been shown to inhibit growth of cancerous cells, mainly by inducing apoptosis [166], and has been shown to be protective against colorectal cancer [167,168]. Whole grain intake is associated with lower body mass index and central adiposity [169], which could have the effect of reducing adiposity-related cancers.

Not all meta-analyses and systematic reviews show uniformly lower cancer risks associated with cereal fiber intake [170,171,172]. However, these meta-analyses focused on breast, endometrial, and renal cancer, which suggests that cereal fiber may be more important for reducing colorectal cancer risk, as discussed above. It should be noted, however, that whole grain intake is associated with significantly lower cancer risk even after adjusting for cereal fiber intake [82]. Thus, the consistent finding of lower risk of total and site-specific cancers associated with whole grain intake may be due to other properties of whole grains [173]. For example, in contrast to the meta-analyses reporting no association between cereal fiber intake and risk of breast or endometrial cancer [170,171], Xiao et al. [30] reported lower breast cancer risk associated with whole grain intake in both categorical and dose-response analyses, and Jacobs et al. [24] reported lower risk of endometrial cancer in highest vs. lowest whole grain intake groups (Table 2). Whole grain foods are a major source of antioxidants and phenolic acids, which can reduce oxidative damage [173]. Whole grain foods contain significant quantities of bioactive compounds that have anticarcinogenic properties [156,157,173,174]. The collective anticarcinogenic impacts of these bioactive compounds likely explain much of the findings from prospective cohort studies [157,173]. 

### 4.2. Strengths and Weaknesses of the Meta-Analyses

A major weakness in the literature summarized in this review is the lack of consistency in the definitions of whole grains and refined grains in studies included in the meta-analyses (Supplementary Tables S2 and S3). Although many studies provided detailed descriptions of foods defined as whole grains [53,71,83,85,91,92,93,94,95,96,98,100,105,108,110,115,121,132,133,134], a number of them did not. For example, many studies included only one whole grain food in the definition of whole grains, such as whole grain bread or whole meal bread [42,43,44,45,46,52,54,65,66,67,68,69,74,75,76,79,80,86,87,88,90,103,106,109,119,138]. Even more problematic, 17 studies provided no definition of whole grains [73,81,97,99,101,104,107,111,112,114,116,120,122,126,127,130,135]. Two cohort studies defined whole grains as foods containing >25% whole grains or bran [82,117], which suggests that whole grain foods could have included a substantial amount of non-whole grain foods. Future research must include more comprehensive definitions of whole grain foods to better understand the association between whole grain intake and cancer risk.

Whereas whole grains are typically defined to include staple grain foods such as bread, cereals, and pasta, many studies have included indulgent grain foods in their definition of refined grains [5]. In the studies used in the meta-analyses presented in Table 3, refined grains have been defined to include cookies, doughnuts, sugar and layer cake [118], sweet buns [95,112,118], pancakes and waffles [83,95,112,117,118], muffins [83,112], and pizza [83,112,117]. These represent very commonly consumed foods and frequently contain high amounts of sugar and/or fat. It is plausible that consuming large amounts of indulgent grain foods may offset any beneficial effects of staple grain foods. It is also important to note that most of the studies used in the meta-analyses did not specifically define refined grains (Supplementary Table S3). Lastly, the number of studies included in the meta-analyses on refined grains was comparatively small. Three of the meta-analyses included only 2 studies, and 1 meta-analysis included only 3 studies. Although one meta-analysis included 18 studies, 14 of the publications used in that meta-analysis did not provide a definition for refined grains (Supplementary Table S3). For these reasons, and relative lack of published data, the associations between refined grain intake and cancer risk must be viewed very cautiously.

On the other hand, refined grain foods are an important source of dietary fiber due to the fact that refined grain consumption is much greater than whole grain consumption [175,176]. Based on National Health and Nutrition Examination Survey data in the United States, for example, 39% of dietary fiber comes from grain foods that contain no whole grain [175]. Thus, refined grains from staple grain foods are an important source of cereal fiber, and cereal fiber consumption has been shown to be associated with reduced risk of cancer [19,162,163]. Research on the distinct associations between consumption of staple and indulgent foods made with refined grains is needed.

It is also important to acknowledge that 6 of the 9 meta-analyses on total cancer mortality relied on essentially the same 6 cohort studies for determination of relative risks [81,82,83,84,85,87]. These cohorts, as well as all others included in the meta-analyses of total cancer mortality, are all from the United States or Europe. Results from these cohorts may not be broadly generalizable to other populations throughout the world. In addition, it is acknowledged that only 1 author performed the search and selection of the meta-analyses included in this review. However, three databases were used in the search, and all selected meta-analyses identified in the initial search were further evaluated by hand-searching their reference lists and examining citation records to find additional meta-analyses. This process yielded 3 additional publications that satisfied inclusion criteria. Thus, the author is confident that the search methodology identified all relevant meta-analyses.

Despite these weaknesses, the meta-analyses reviewed have some notable strengths. With two exceptions, all meta-analyses reported no publication bias that might confound interpretation of the results. The only meta-analysis that reported a significant Egger’s test was that of Zhang et al. [32] on esophageal cancer. Jacobs et al. [24] did not report data on bias or heterogeneity. Most of the meta-analyses reported statistically significant I^2^, which suggests that variation across studies was not due to chance. The considerable differences in definitions of whole and refined grains may also have contributed to the significant heterogeneity across studies. 

## 5. Conclusions

Meta-analyses consistently show that whole grain consumption is associated with lower risk of total cancer mortality. Risk reductions for the highest intakes of whole grains ranged between 5% and 12%. In dose-response analyses, each 30 g/day intake of whole grains was associated with a ~7% lower risk of cancer mortality. For site-specific cancers, meta-analyses indicate that whole grain intake is consistently associated with lower cancer risk, with the strongest evidence for colorectal, gastric, pancreatic, and esophageal cancers. The only cancer for which whole grain intake was not associated with lower risk was prostate cancer. Overall, these meta-analyses of cohort and case-control studies support the recommendations for increased whole grain consumption [2,3].

Refined grain intake may reduce the risk of total cancer, but the meta-analysis included only 2 studies. High intake of refined grain may increase risk of gastric cancer, but these conclusions must be viewed skeptically due to the weaknesses described above. Considerably more research is necessary on the association between refined grain intake and cancer risk before definitive conclusions can be drawn and evidence-based dietary guidelines established.

## Figures and Tables

**Table 1:** Whole grain intake and risk of cancer mortality *: Results from meta-analyses of observational cohort and case-control studies.

Meta-Analysis	Highest vs. Lowest Intakes		Dose Response	
	Number of Cohorts or Case-Control Studies Included [References]	Relative Risk or Odds Ratio (95% CI)	Number of Cohorts or Case-Control Studies Included [References]	Relative Risk or Odds Ratio (95% CI)
Jacobs et al., 1998 [24]	45 [41,42,43,44,45,46,47,48,49,50,51,52,53,54,55,56,57,58,59,60,61,62,63,64,65,66,67,68,69,70,71,72,73,74,75,76,77,78,79,80]	0.66 (0.60–0.72)		
Aune et al., 2016 [20]	6 [81,82,83,84,85]	0.89 (0.82–0.96)	6 [81,82,83,84,85]	0.85 (0.80–0.91) 90 g/day
Benisi-Kohansel et al., 2016 [21]	7 [81,82,83,85,86,87]	0.94 (0.91–0.98)	3 [82,83,85]	0.90 (0.83–0.98) 90 g/day
Chen et al., 2016 [22]	8 [81,82,83,84,85,87,88]	0.89 (0.84–0.95)	6 [81,82,83,84,85]	0.82 (0.69–0.86) 50 g/day
Wei et al., 2016 [29]	8 [81,82,83,84,85,87]	0.89 (0.82–0.96)	7 [81,82,83,84,85]	0.91 (0.84–0.98) 90 g/day
Zong et al., 2016 [33]	10 [82,83,84,85,87,88]	0.88 (0.83–0.94)	10 [82,83,84,85,87,88]	0.80 (0.72–0.89) 70 g/day 0.85 (0.76–0.94) 50 g/day 0.89 (0.79–0.99) 30 g/day 0.96 (0.91–1.01) 10 g/day
Zhang et al., 2018 [31]	14 [82,85,87,89,90,91,92,93,94,95,96,97]	0.94 (0.87–1.01)	14 [82,85,87,89,90,91,92,93,94,95,96,97]	0.97 (0.95–0.99) 28 g/day
Reynolds et al., 2019 [26]	5 [81,82,83,84,85]	0.84 (0.76–0.92)	7 [81,82,83,84,85]	0.95 (0.93–0.97) 15 g/day

CI = confidence interval; * all meta-analyses reported total cancer mortality except for the case-control meta-analysis of Jacobs et al., who reported total cancer risk for multiple sites combined. Unfilled field indicates that no meta-analyses were performed. g/day refers to the dose of whole grain intake associated with the corresponding relative risk or odds ratio in the dose-response analysis.

**Table 2:** Whole grain intake and risk of site-specific cancer: Results from meta-analyses of observational cohort and case-control studies.

Meta-Analysis	Highest vs. Lowest Intakes		Dose Response		Cancer Site
	Number of Cohorts or Case-Control Studies Included [References]	Relative Risk or Odds Ratio (95% CI)	Number of Cohorts or Case-Control Studies Included [References]	Relative Risk or Odds Ratio (95% CI)	
Jacobs et al., 1998 [24]	7 [41,46,61,68,69,71,75]	0.79 (0.69–0.89)			Colorectal
Aune et al., 2011 [19]	4 [95,98,99,100]	0.79 (0.72–0.86)	6 [95,98,99,100,101]	0.83 (0.78–0.89)	Colorectal 90 g/day
Vieira et al., 2017 [28]			6 [94,95,100,102]	0.83 (0.79–0.89)	Colorectal 90 g/day
Schwingshackl et al., 2018 [16]	10 [90,94,95,99,100,101,102,103,104]	0.88 (0.83–0.94)	9 [90,94,95,99,100,101,102,104]	0.95 (0.93–0.97)	Colorectal 30 g/day
Reynolds et al., 2019 [26]	7 [90,94,95,98,101,102,105]	0.87 (0.79–0.96)	8 [90,94,95,98,101,102,105]	0.97 (0.95–0.99)	Colorectal 15 g/day
Zhang et al., 2020 [32]	25 [46,68,71,90,94,95,98,99,100,101,105,106,107,108,109,110,111,112]	0.89 (0.84–0.93)			Colorectal
Aune et al., 2011 [19]	5 [95,98,100,105,112]	0.82 (0.72–0.92)	4 [95,98,100,105]	0.86 (0.79–0.94)	Colon 90 g/day
Vieira et al., 2017 [28]			4 [94,95,100,105]	0.82 (0.73–0.92)	Colon 90 g/day
Schwingshackl et al., 2018 [16]	7 [90,94,95,100,104,105,112]	0.85 (0.77–0.93)	6 [90,94,95,100,104]	0.97 (0.95–0.99)	Colon 30 g/day
Aune et al., 2011 [19]	3 [95,98,100]	0.80 (0.59–1.07)	3 [95,98,100]	0.80 (0.56–1.14)	Rectal 90 g/day
Vieira et al., 2017 [28]			3 [94,95,100]	0.81 (0.54–1.20)	Rectal 90 g/day
Schwingshackl et al., 2018 [16]	5 [90,94,95,100,104]	0.80 (0.64–0.98)	5 [90,94,95,100,104]	0.94 (0.88–1.01)	Rectal 30 g/day
Jacobs et al., 1998 [24]	7 [42,43,54,59,74,76,79]	0.57 (0.47–0.67)			Gastric
Wang et al., 2020 [35]	5 [42,43,113,114,115]	0.87 (0.79–0.95)			Gastric
Xu et al., 2019 [36]	3 [116,117,118]	0.61 (0.40–0.83)			Gastric
Zhang et al., 2020 [32]	12 [42,43,54,79,113,115,116,117,118,119,120]	0.64 (0.53–0.79)			Gastric
Jacobs et al., 1998 [24]	4 [45,52,65,67]	0.70 (0.54–0.86)			Pancreatic
Lei et al., 2016 [25]	5 [24,121,122,123,124]	0.76 (0.64–0.91)			Pancreatic
Wang et al., 2015 [34]	8 [91,96,125,126,127,128,129,130]	1.13 (0.98–1.30)			Prostate
Reynolds et al., 2019 [26]	3 [91,96,127]	1.10 (1.02–1.19)	2 [91,96,127]	1.02 (0.98–1.05)	Prostate 15 g/day
Jacobs et al., 1998 [24]	2 [56,64]	0.86 (0.67–1.05)			Breast
Xiao et al., 2018 [30]	11 [56,64,92,125,131,132,133,134,135,136,137]	0.84 (0.74–0.96)	6 [92,131,132,135,136,137]	0.83 (0.73–0.93)	Breast 50 g/day
Jacobs et al., 1998 [24]	2 [47,80]	0.52 (0.09–0.95)			Esophageal
Zhang et al., 2020 [32]	7 [117,138,139,140,141,142]	0.54 (0.44–0.67)			Esophageal
Jacobs et al., 1998 [24]	4 [48,58,66,77]	0.57 (0.38–0.76)			Oral
Jacobs et al., 1998 [24]	2 [44,51]	0.67 (0.48–0.86)			Brain
Jacobs et al., 1998 [24]	3 [53,55,63]	0.55 (0.41–0.69)			Endometrial
Jacobs et al., 1998 [24]	2 [50,73]	0.41 (0.37–0.45)			Non-Hodgkin’s lymphoma

CI = confidence interval. Unfilled fields indicate that no meta-analyses were performed. g/day refers to the dose of whole grain intake associated with the corresponding relative risk or odds ratio in the dose-response analysis.

**Table 3:** Refined grain intake and cancer risk: Results from meta-analyses of observational cohort and case-control studies.

Meta-Analysis	Highest vs. Lowest Intakes		Dose Response		Cancer
	Number of Cohorts or Case-Control Studies Included [References]	Relative Risk or Odds Ratio (95% CI)	Number of Cohorts or Case-Control Studies Included [References]	Relative Risk or Odds Ratio (95% CI)	
Aune et al., 2016 [20]			2 [83,85]	0.94 (0.90–0.99)	Total cancer 90 g/day
Schwingshackl et al., 2018 [16]	2 [95,103]	1.46 (0.80–2.67)			Colorectal
Schwingshackl et al., 2018 [16]	2 [95,112]	1.27 (1.02–1.57)			Colon
Xu et al., 2019 [36]	3 [116,117,118]	1.65 (1.36–1.94)			Gastric
Wang et al., 2020 [35]	18 [54,59,113,114,116,118,143,144,145,146,147,148,149,150,151,152]	1.36 (1.21–1.54)			Gastric

CI = confidence interval. Unfilled fields indicate that no meta-analyses were performed. g/day refers to the dose of whole grain intake associated with the corresponding relative risk or odds ratio in the dose-response analysis.

## Supplementary Materials

The following are available online at https://www.mdpi.com/2072-6643/12/12/3756/s1, Table S1: Characteristics of the meta-analyses included in this review; Table S2: Characteristics of studies included in the meta-analyses on the association between whole grain intake and cancer risk; Table S3: Characteristics of studies included in the meta-analyses on the association between refined grain intake and cancer risk.
