# Supplementary material for: Whole Grains, Refined Grains, and Cancer Risk: A Systematic Review of Meta-Analyses of Observational Studies
Source: Nutrients. 2020 Dec 7;12(12):3756. doi: 10.3390/nu12123756 (PMC7762239; doi:10.3390/nu12123756)
Supplement: Supplementary file 1 [file nutrients-12-03756-s001.zip › Supplementary Table 1.docx]

**Supplementary Table 1.** Characteristics of the meta-analyses included in this review

| **Meta-analysis** | **Cancer outcome or site** | **Number of cohorts or case-control studies included**  **(number of participants)** | **Publication bias assessment** | **Heterogeneity (I^2^ )** |
| --- | --- | --- | --- | --- |
| **Meta-analyses on Whole Grains** | | | | |
| Aune et al. 2016 [1] | Total cancer mortality | 6 cohorts from 5 publications  (640,065) | Egger’s: P = 0.44 | H-L: I^2^ = 72%,  P = 0.003  D-R: I^2^ = 32%,  P = 0.16 |
| Benisi-Kohansel et al. 2016 [2] | Total cancer mortality | 7 cohorts from 6 publications  (564,644) | Begg’s: P = 0.35  Egger’s: P = 0.29 | H-L: I^2^ = 0%,  P = 0.51  D-R: not reported |
| Chen et al. 2016 [3] | Total cancer mortality | 8 cohorts from 7 publications  (684,890) | Begg’s: P = 0.71  Egger’s: P = 0.75 | H-L: I^2^ = 53.6%,  P = 0.04  D-R: I^2^ = 83.5%,  P < 0.001 |
| Wei et al. 2016 [4] | Total cancer mortality | 8 cohorts from 6 publications  (673,912) | Begg’s: P = 0.17  Egger’s: P = 0.96 | H-L: 63.6%,  P = 0.007  D-R: 78.4%,  P < 0.001 |
| Reynolds et al. 2019 [5] | Total cancer mortality | 7 cohorts from 5 publications  (640,065) | ROBIS: Not serious | H-L: 78.3%,  P = 0.001  D-R: 82.0%,  P = 0.005 |
| Zhang et al. 2018 [6] | Total cancer mortality | 14 cohorts from 12 publications  (834,935) | Begg’s: P = 0.17  Egger’s: P = 0.60 | H-L: 69.4%,  P < 0.001  D-R: not reported |
| Zong et al. 2016 [7] | Total cancer mortality | 10 cohorts from 6 publications and 2 unpublished NHANES data sets  (701,130) | Egger’s: P = 0.64 | H-L: 54%, P = 0.02  D-R: not reported |
| Jacobs et al. 1998 [8] | Total cancer risk  (multiple sites) | 45 case-control studies  (14,618 cases, 29,050 controls) | Not reported | Not reported |
| Aune et al. 2011 [9] | Colorectal | 8 cohorts from 7 publications  (955,046) | Begg’s: P = 1.00  Egger’s: P = 0.54 | H-L: 0%, P = 0.98  D-R: 18.0%, P = 0.30 |
| Aune et al. 2011 [9] | Colon | 5 cohorts from 5 publications  (689,371) | Begg’s: P = 1.00  Egger’s: P = 0.54 | H-L: 23%, P = 0.27  D-R: 0%, P = 0.42 |
| Aune et al. 2011 [9] | Rectal | 3 cohorts from 3 publications  (606,863) | Begg’s: P = 1.00  Egger’s: P = 0.54 | H-L: 58%, P = 0.10  D-R: 91%, P < 0.001 |
| Vieira et al. 2017 [10] | Colorectal | 6 cohorts from 5 publications  (822,901) | Egger’s: P > 0.05 | D-R: 0%, P = 0.30 |
| Vieira et al. 2017 [10] | Colon | 4 cohorts from 4 publications  (792,207) | Egger’s: P > 0.05 | D-R: 0%, P = 0.49 |
| Vieira et al. 2017 [10] | Rectal | 3 cohorts from 3 publications  (578,155) | Egger’s: P > 0.05 | D-R: 91%, P < 0.001 |
| Reynolds et al. 2019 [5] | Colorectal | 8 cohorts from 7 publications  (1,010,729) | ROBIS: Not serious | H-L: 51.9%,  P = 0.053  D-R: 45%, P = 0.009 |
| Schwingshackl et al. 2018 [11] | Colorectal | 10 cohorts from 9 publications  (970,927) | Egger’s: P = 0.07 | H-L: 35%, P = 0.13  D-R: 58%, P = 0.02 |
| Schwingshackl et al. 2018 [11] | Colon | 7 cohorts from 7 publications  (949,521) | Not assessed | H-L: 0%, P = 0.59  D-R: 45%, P = 0.009 |
| Schwingshackl et al. 2018 [11] | Rectal | 5 cohorts from 5 publications  (765,229) | Not assessed | H-L: 0%, P = 0.73  D-R: 73%, P = 0.006 |
| Reynolds et al. 2019 [5] | Prostate | 3 cohorts from 3 publications  (84,753) | ROBIS: Not serious | H-L: 0%, P = 0.50  D-R: Not reported |
| Xu et al. 2019 [12] | Gastric | 1 cohort study  (34,651)  2 case-control studies  (1,019 cases, 2,939 controls) | Begg’s: P = 0.84  Egger’s: P = 0.69 | H-L: 52.5%, P = 0.12 |
| Wang et al. 2020 [13] | Gastric | 1 cohort study  (133,163)  4 case-control studies  (1,284 cases, 2,099 controls) | Egger’s: P > 0.05 | H-L: 6.6%, P = 0.37 |
| Lei et al. 2016 [14] | Pancreatic | 1 cohort study  (27,111)  4 case-control studies  (2,389 cases, 14,334 controls) | Not reported | H-L: 11.7%, P = 0.34 |
| Wang et al. 2015 [15] | Prostate | 3 cohort studies from 3 publications  (84,753)  5 case-control studies  (1,771 cases, 5,191 controls) | Begg’s: P = 1.00  Egger’s: P = 0.48 | H-L: 52.5%, P = 0.04 |
| Xiao et al. 2018 [16] | Breast | 4 cohort studies from 4 publications  (84,753)  7 case-control studies  (1,771 cases, 5,191 controls) | Begg’s: P = 30  Egger’s: P = 0.31 | H-L: 63.8%,  P = 0.002  D-R: 70.5%,  P = 0.005 |
| Zhang et al. 2020 [17] | Esophageal | 2 cohort studies from 2 publications  (148,463)  5 case-control studies from 4 publications  (942 cases, 2,082 controls) | Egger’s: P < 0.05 | H-L: 27.7%,  P = 0.217 |
| Zhang et al. 2020 [17] | Colorectal | 17 cohort studies from 11 publications  (1,395,465)  8 case-control studies from 7 publications  (3,760 cases, 4,257 controls) | Egger’s: P > 0.05 | H-L: 38.2%,  P = 0.029 |
| Zhang et al. 2020 [17] | Gastric | 3 cohort studies from 2 publications  (1,004,696)  9 case-control studies from 9 publications  (4,047 cases, 9,364 controls) | Egger’s: P > 0.05 | H-L: 78.2%,  P = 0.000 |
| Jacobs et al. 1998 [8] | Gastric | 7 case-control studies from 7 publications  (2,124 cases, 6,234 controls) | Not reported | Not reported |
| Jacobs et al. 1998 [8] | Colorectal | 7 case-control studies from 7 publications  (4,434 cases, 7,668 controls) | Not reported | Not reported |
| Jacobs et al. 1998 [8] | Pancreatic | 4 case-control studies from 4 publications  (1,067 cases, 1,592 controls) | Not reported | Not reported |
| Jacobs et al. 1998 [8] | Breast | 2 case-control studies from 2 publications  (1,125 cases, 1,599 controls) | Not reported | Not reported |
| Jacobs et al. 1998 [8] | Oral | 4 case-control studies from 4 publications  (1,414 cases, 2,953 controls) | Not reported | Not reported |
| Jacobs et al. 1998 [8] | Esophageal | 2 case-control studies from 2 publications  (380 cases, 623 controls) | Not reported | Not reported |
| Jacobs et al. 1998 [8] | Brain | 2 case-control studies from 2 publications  (622 cases, 824 controls) | Not reported | Not reported |
| Jacobs et al. 1998 [8] | Endometrial | 3 case-control studies from 3 publications  (812 cases, 1,289 controls) | Not reported | Not reported |
| Jacobs et al. 1998 [8] | Non-Hodgkin’s Lymphoma | 2 case-control studies from 2 publications  (1,037 cases, 1,558 controls) | Not reported | Not reported |
| **Meta-analyses on Refined Grains** | | | | |
| Aune et al. 2016 [1] | Total cancer mortality | 2 cohorts from 2 publications  (145,397) | Not reported | D-R: 0%, P = 0.60 |
| Schwingshackl et al. 2018 [11] | Colorectal | 2 cohorts from 2 publications  (72,432) | Not reported | H-L: 71%, P = 0.06 |
| Schwingshackl et al. 2018 [11] | Colon | 2 cohorts from 2 publications  (110,733) | Not reported | H-L: 0%, P = 0.89 |
| Xu et al. 2019 [12] | Gastric | 1 cohort study from 1 publication (34,651)  2 case-control studies from 2 publications  (1,109 cases, 3,989 controls) | Not reported | H-L: 0%, P = 0.75 |
| Wang et al. 2020 [13] | Gastric | 1 cohort study from 1 publication (7,925)  17 case-control studies from 16 publications  (5,250 cases, 11,288 controls) | Egger’s: P > 0.05 | H-L: 56.5%,  P = 0.002 |

H-L: Highest vs. Lowest intake comparisons; D-R: Dose-Response analyses; Egger’s test: [18]; Begg’s test: [19]; ROBIS: [20]; I^2^ statistic: [21]

**References**

1. Aune, D.; Keum, N.; Giovannucci, E.; Fadnes, L.T.; Boffetta, P.; Greenwood, D.C.; Tonstad, S.; Vatten, L.J.; Riboli, E.; Norat, T. Whole grain consumption and risk of cardiovascular disease, cancer, and all cause and cause specific mortality: systematic review and dose-response meta-analysis of prospective studies. *BMJ* **2016**, *353*, i2716, doi:10.1136/bmj.i2716.

2. Benisi-Kohansal, S.; Saneei, P.; Salehi-Marzijarani, M.; Larijani, B.; Esmaillzadeh, A. Whole-Grain Intake and Mortality from All Causes, Cardiovascular Disease, and Cancer: A Systematic Review and Dose-Response Meta-Analysis of Prospective Cohort Studies. *Adv Nutr* **2016**, *7*, 1052-1065, doi:10.3945/an.115.011635.

3. Chen, G.C.; Tong, X.; Xu, J.Y.; Han, S.F.; Wan, Z.X.; Qin, J.B.; Qin, L.Q. Whole-grain intake and total, cardiovascular, and cancer mortality: a systematic review and meta-analysis of prospective studies. *Am J Clin Nutr* **2016**, *104*, 164-172, doi:10.3945/ajcn.115.122432.

4. Wei, H.; Gao, Z.; Liang, R.; Li, Z.; Hao, H.; Liu, X. Whole-grain consumption and the risk of all-cause, CVD and cancer mortality: a meta-analysis of prospective cohort studies. *Br J Nutr* **2016**, *116*, 514-525, doi:10.1017/S0007114516001975.

5. Reynolds, A.; Mann, J.; Cummings, J.; Winter, N.; Mete, E.; Te Morenga, L. Carbohydrate quality and human health: a series of systematic reviews and meta-analyses. *Lancet* **2019**, *393*, 434-445, doi:10.1016/S0140-6736(18)31809-9.

6. Zhang, B.; Zhao, Q.; Guo, W.; Bao, W.; Wang, X. Association of whole grain intake with all-cause, cardiovascular, and cancer mortality: a systematic review and dose-response meta-analysis from prospective cohort studies. *Eur J Clin Nutr* **2018**, *72*, 57-65, doi:10.1038/ejcn.2017.149.

7. Zong, G.; Gao, A.; Hu, F.B.; Sun, Q. Whole Grain Intake and Mortality From All Causes, Cardiovascular Disease, and Cancer: A Meta-Analysis of Prospective Cohort Studies. *Circulation* **2016**, *133*, 2370-2380, doi:10.1161/CIRCULATIONAHA.115.021101.

8. Jacobs, D.R., Jr.; Marquart, L.; Slavin, J.; Kushi, L.H. Whole-grain intake and cancer: an expanded review and meta-analysis. *Nutr Cancer* **1998**, *30*, 85-96, doi:10.1080/01635589809514647.

9. Aune, D.; Chan, D.S.; Lau, R.; Vieira, R.; Greenwood, D.C.; Kampman, E.; Norat, T. Dietary fibre, whole grains, and risk of colorectal cancer: systematic review and dose-response meta-analysis of prospective studies. *BMJ* **2011**, *343*, d6617, doi:10.1136/bmj.d6617.

10. Vieira, A.R.; Abar, L.; Chan, D.S.M.; Vingeliene, S.; Polemiti, E.; Stevens, C.; Greenwood, D.; Norat, T. Foods and beverages and colorectal cancer risk: a systematic review and meta-analysis of cohort studies, an update of the evidence of the WCRF-AICR Continuous Update Project. *Ann Oncol* **2017**, *28*, 1788-1802, doi:10.1093/annonc/mdx171.

11. Schwingshackl, L.; Schwedhelm, C.; Hoffmann, G.; Knuppel, S.; Laure Preterre, A.; Iqbal, K.; Bechthold, A.; De Henauw, S.; Michels, N.; Devleesschauwer, B., et al. Food groups and risk of colorectal cancer. *Int J Cancer* **2018**, *142*, 1748-1758, doi:10.1002/ijc.31198.

12. Xu, Y.; Yang, J.; Du, L.; Li, K.; Zhou, Y. Association of whole grain, refined grain, and cereal consumption with gastric cancer risk: A meta-analysis of observational studies. *Food Sci Nutr* **2019**, *7*, 256-265, doi:10.1002/fsn3.878.

13. Wang, T.; Zhan, R.; Lu, J.; Zhong, L.; Peng, X.; Wang, M.; Tang, S. Grain consumption and risk of gastric cancer: a meta-analysis. *Int J Food Sci Nutr* **2020**, *71*, 164-175, doi:10.1080/09637486.2019.1631264.

14. Lei, Q.; Zheng, H.; Bi, J.; Wang, X.; Jiang, T.; Gao, X.; Tian, F.; Xu, M.; Wu, C.; Zhang, L., et al. Whole Grain Intake Reduces Pancreatic Cancer Risk: A Meta-Analysis of Observational Studies. *Medicine (Baltimore)* **2016**, *95*, e2747, doi:10.1097/MD.0000000000002747.

15. Wang, R.J.; Tang, J.E.; Chen, Y.; Gao, J.G. Dietary fiber, whole grains, carbohydrate, glycemic index, and glycemic load in relation to risk of prostate cancer. *Onco Targets Ther* **2015**, *8*, 2415-2426, doi:10.2147/OTT.S88528.

16. Xiao, Y.; Ke, Y.; Wu, S.; Huang, S.; Li, S.; Lv, Z.; Yeoh, E.K.; Lao, X.; Wong, S.; Kim, J.H., et al. Association between whole grain intake and breast cancer risk: a systematic review and meta-analysis of observational studies. *Nutr J* **2018**, *17*, 87, doi:10.1186/s12937-018-0394-2.

17. Zhang, X.F.; Wang, X.K.; Tang, Y.J.; Guan, X.X.; Guo, Y.; Fan, J.M.; Cui, L.L. Association of whole grains intake and the risk of digestive tract cancer: a systematic review and meta-analysis. *Nutr J* **2020**, *19*, 52, doi:10.1186/s12937-020-00556-6.

18. Egger, M.; Davey Smith, G.; Schneider, M.; Minder, C. Bias in meta-analysis detected by a simple, graphical test. *BMJ* **1997**, *315*, 629-634, doi:10.1136/bmj.315.7109.629.

19. Begg, C.B.; Mazumdar, M. Operating characteristics of a rank correlation test for publication bias. *Biometrics* **1994**, *50*, 1088-1101.

20. Whiting, P.; Savovic, J.; Higgins, J.P.; Caldwell, D.M.; Reeves, B.C.; Shea, B.; Davies, P.; Kleijnen, J.; Churchill, R.; group, R. ROBIS: A new tool to assess risk of bias in systematic reviews was developed. *J Clin Epidemiol* **2016**, *69*, 225-234, doi:10.1016/j.jclinepi.2015.06.005.

21. Higgins, J.P.; Thompson, S.G.; Deeks, J.J.; Altman, D.G. Measuring inconsistency in meta-analyses. *BMJ* **2003**, *327*, 557-560, doi:10.1136/bmj.327.7414.557.
