# Supplementary material for: Whole Grains, Refined Grains, and Cancer Risk: A Systematic Review of Meta-Analyses of Observational Studies
Source: Nutrients. 2020 Dec 7;12(12):3756. doi: 10.3390/nu12123756 (PMC7762239; doi:10.3390/nu12123756)
Supplement: Supplementary file 1 [file nutrients-12-03756-s001.zip › Supplementary Table 2.docx]

**Supplementary Table 2.** Characteristics of studies included in the meta-analyses on the association between whole grain intake and cancer risk

| **Study** | **Population** | **Dietary Assessment** | **Definition of whole grain** | **Meta-analysis included in** | **Cancer outcome or site** |
| --- | --- | --- | --- | --- | --- |
| Huang et al. 2015 [1] | NIH-AARP Diet and Health Study (United States)  367,442 men and women | FFQ | Foods containing >25% whole grains and/or bran | Aune 2016  Benisi-Kohansel 2016  Chen 2016  Reynolds 2019  Wei 2016  Zhang 2018  Zong 2016 | Total cancer mortality |
| Wu et al. 2015 [2] | Nurses’ Health Study I 74,341 women  Health Professionals Follow-up Study  43,744 men  (both United States) | FFQ | Whole wheat and whole wheat flour, whole oats and whole oat flour, whole cornmeal and whole corn flour, whole rye and whole rye flour, whole barley, bulgur, buckwheat, brown rice and brown rice  flour, popcorn, amaranth, psyllium | Aune 2016  Benisi-Kohansel 2016  Chen 2016  Reynolds 2019  Wei 2016  Zhang 2018  Zong 2016 | Total cancer mortality |
| Jacobs et al. 2007 [3] | Iowa Women’s Health Study (United States)  27,312 women | FFQ | Dark bread, cold breakfast cereal, brown rice, popcorn, wheat germ, bran, cooked oatmeal, and other grains (e.g., bulgar, kasha,  and couscous) | Aune 2016  Benisi-Kohansel 2016  Chen 2016  Reynolds 2019  Wei 2016  Zong 2016 | Total cancer mortality |
| Johnsen et al. 2015 [4] | HELGA cohort (Denmark, Norway, Sweden)  120,010 men and women | FFQ | Whole grain breakfast cereals, non-white  bread and crisp bread | Aune 2016  Chen 2016  Reynolds 2019  Wei 2016  Zong 2016 | Total cancer mortality |
| Buil-Cosiales et al. 2014 [5] | PREDIMED trial (Spain)  7,216 men and women | FFQ | Not defined | Aune 2016  Benisi-Kohansel 2016  Chen 2016  Reynolds 2019  Wei 2016 | Total cancer mortality |
| Jacobs et al. 2001 [6] | Norwegian County Study (Norway)  33,848 men and women | FFQ | Whole grain bread | Benisi-Kohansel 2016  Chen 2016  Wei 2016  Zhang 2018  Zong 2016 | Total cancer mortality |
| Key et al. 1996 [7] | Vegetarians and health-conscious adults (United Kingdom)  10,771 men and women | FFQ | Whole meal bread | Chen 2016  Zong 2016 | Total cancer mortality |
| Appleby et al. 2002 [8] | Health Food Shoppers Study (United Kingdom)  11,000 men and women | Diet and Lifestyle Questionnaire | Whole meal bread | Benisi-Kohansel 2016 | Total cancer mortality |
| Aarestrup et al. 2012 [9] | Diet, Cancer and Health Study (Denmark)  29,537 women | FFQ | Rye bread, whole grain bread, oatmeal and muesli, and crispbread | Zhang 2018 | Total cancer mortality |
| Jacobs et al. 1999 [10] | Iowa Women’s Health Study (United States) 99,826 women | FFQ | Dark bread, cold breakfast cereal, brown rice, popcorn, wheat germ, bran, cooked oatmeal, and other grains (e.g., bulgar, kasha,  and couscous) | Zhang 2018 | Total cancer mortality |
| Larsson et al. 2005 [11] | Swedish Mammography Cohort (Sweden)  61,433 women | FFQ | Whole grain rye bread (such as Wasa bread), soft whole grain bread, porridge, and cold  breakfast cereals | Aune 2011  Schwingshackl 2018  Reynolds 2019  Vieira 2017  Zhang 2018  Zhang 2020 | Colorectal  Colon  Rectal |
| Schatzkin et al. 2007 [12] | NIH-AARP Diet and Health Study (United States)  489,611 men and women | FFQ | One serving of whole grain was defined on the basis of standard  portion sizes developed by the USDA, such as one slice of wholegrain  bread, one cup of ready-to-eat whole-grain cereal, or 0.5 cups of cooked whole grains | Aune 2011, Schwingshackl 2018, Reynolds 2019  Vieira 2017  Zhang 2020 | Colorectal  Colon  Rectal |
| Kyro et al. 2013 [13] | HELGA cohort (Denmark, Norway, Sweden)  108,000 men and women | FFQ | Rye, wheat, oats, barley, rice, millet, corn/maize (dried), triticale, and sorghum/durra. No cut-off limit was set for the whole-grain  content of a product. | Schwingshackl 2018  Reynolds 2019  Vieira 2017  Zhang 2020 | Colorectal  Colon  Rectal |
| Egeberg et al. 2010 [14] | Diet, Cancer and Health Study (Denmark)  58,819 men and women | FFQ | Whole grain products from breads and cereals, including whole grain bread and whole grain rye bread, and oatmeal | Aune 2011  Reynolds 2019  Zhang 2020 | Colorectal  Colon  Rectal |
| Bakken et al. 2016 [15] | Norwegian Women and Cancer Study (Norway)  78,254 women | FFQ | Whole grain bread | Schwingshackl 2018  Zhang 2020 | Colorectal  Colon  Rectal |
| Fung et al. 2010 [16] | Nurses’ Health Study I 87,256 women  Health Professionals Follow-up Study  45,490 men  (both United States) | FFQ | Not defined | Aune 2011  Reynolds 2019  Schwingshackl 2018  Zhang 2020 | Colorectal |
| McCarl et al. 2006 [17] | Iowa Women’s Health Study (United States) 35,197 women | FFQ | Not defined | Aune 2011 Schwingshackl 2018  Zhang 2020 | Colorectal |
| Pietinen et al. 1999 [18] | Alpha-Tocopherol, Beta-Carotene Cancer Prevention Study (Finland)  27,111 men (smokers) | FFQ | Breads, porridges, and whole-grain breakfast cereals | Reynolds 2019  Schwingshackl 2018  Vieira 2017 | Colorectal |
| Peters et al. 1989 [19] | Case-control (United States) – men only  147 cases, 147 controls | Structured Questionnaire | Whole grain bread | Jacobs 1998  Zhang 2020 | Colorectal |
| Centonze et al. 1994 [20] | Case-control (Italy)  119 cases (66 men, 53 women)  119 controls (64 men, 55 women) | FFQ | Whole meal bread | Jacobs 1998  Zhang 2020 | Colorectal |
| Slattery et al. 1997 [21] | Case-control (United States)  1,993 cases (1,099 men, 894 women)  2,410 controls (1,290 men, 1,120 women) | FFQ | Whole wheat and high fiber breads, bran muffins, whole grain cereals, brown rice, kasha, and high fiber crackers | Jacobs 1998  Zhang 2020 | Colorectal |
| Sanjoaquin et al. 2004 [22] | Oxford Vegetarian Study (United Kingdom)  4,162 men  6,836 women | FFQ | Brown bread | Schwingshackl 2018 | Colorectal |
| Vulcan et al. 2015 [23] | Malmo Diet and Cancer Study (Sweden)  10,987 men  16,944 women | FFQ and 7-d modified diet history | Not defined | Schwingshackl 2018 | Colorectal |
| Angelo et al. 2016 [24] | Case-control (Brazil)  169 cases (418 men, 72 women)  101 controls (77 men, 24 women) | FFQ | Not defined | Zhang 2020 | Colorectal |
| Williams et al. 2009 [25] | Case-control (North Carolina Colon Cancer Study – Phase II, United States)  945 cases (535 men, 410 women)  959 controls (571 men, 388 women) | FFQ | Not defined | Zhang 2020 | Colorectal |
| Abu Mweis et al. 2015 [26] | Case-control (Jordan)  167 cases (79 men, 88 women)  240 controls (108 men, 132 women) | FFQ | Whole bread | Zhang 2020 | Colorectal |
| Tayyem et al. 2016 [27] | Case-control (Jordan)  220 cases (116 men, 104 women)  281 controls (132 men, 149 women) | FFQ | Whole wheat bread | Zhang 2020 | Colorectal |
| He et al. 2019 [28] | Nurses’ Health Study 90,869 women  Health Professionals Follow-up Study  47,924 men  (both United States) | FFQ | Whole wheat and whole wheat flour, whole oats and whole oat flour, whole cornmeal and whole corn flour, whole rye and whole rye flour, whole barley, bulgur, buckwheat, brown rice and brown rice  flour, popcorn, amaranth, psyllium | Zhang 2020 | Colorectal |
| Um et al.2019 [29] | Cancer Prevention Study II Nutrition Cohort (United State)  50,118 men  62,031 women | FFQ | Dark bread, whole grain cold breakfast cereals (≥ 50% whole  grain), cooked oatmeal/oat bran and other cooked breakfast cereals, other grains (e.g., bulgar, kasha, couscous, etc.),  brown rice, popcorn, and oat bran, wheat germ and other  bran added to food | Zhang 2020 | Colorectal |
| Bidoli et al. 1992 [30] | Case-control (Italy)  248 cases (155 men, 93 women)  699 controls (551 men, 148 women) | FFQ | Whole grain bread and pasta | Jacobs 1998 | Colorectal |
| Tuyns et al. 1988 [31] | Case-control (Belgium)  818 cases, 2,851 controls  (sex not indicated) | FFQ | Whole meal bread | Jacobs 1998 | Colorectal |
| La Vecchia et al. 1988 [32] | Case-control (Italy)  575 cases (290 men, 285 women)  778 controls (493 men, 285 women) | FFQ | Whole grain bread and pasta | Jacobs 1998 | Colorectal |
| Pickle et al. 1984 [33] | Case-control (United States)  86 cases (40 men, 46 women)  176 controls (89 men, 87 women) | FFQ | Whole grain (whole wheat or pumpernickel) breads, muffins, or rolls | Jacobs 1998 | Colorectal |
| McCullough et al. 2003 [34] | Cancer Prevention Study II Nutrition Cohort (United States)  133,163 men and women | FFQ | High fiber, bran or granola cereals, shredded wheat,  highly fortified cereal (such as Product 19, Total or Most);  cooked cereals;  dark breads such as whole wheat, rye, pumpernickel;  corn bread, corn muffins, corn tortillas or grits | Aune 2011  Schwingshackl 2018  Reynolds 2019  Vieira 2017  Zhang 2020 | Colon |
| Wu et al. 2004 [35] | Health Professionals Follow-up Study (United States)  47,300 men | FFQ | Not defined | Aune 2011, Schwingshackl 2018  Zhang 2020 | Colon |
| La Vecchia et al. 1988 [36]  (data from 1987 publication) | Case-control (Italy)  206 cases (132 men, 74 women), 474 controls (321 men, 153 women) | FFQ | Whole grain bread or pasta | Jacobs 1998  Wang 2020  Zhang 2020 | Gastric |
| Boeing et al. 1991 [37] | Case-control (Germany)  143 cases, 579 controls  (sex not indicated) | FFQ | Whole meal bread | Jacobs 1998  Wang 2020  Zhang 2020 | Gastric |
| Boeing et al. 1991 [38] | Case-control (Poland)  741 cases (520 men, 221 women), 741 controls (520 men, 221 women) | FFQ | Nonwhite bread | Jacobs 1998  Wang 2020  Zhang 2020 | Gastric |
| McCullough et al. 2001 [39] | Cancer Prevention Study II (United States)  436,654 men  533,391 women | FFQ | Brown rice/whole wheat/barley, bran/corn muffins, oatmeal/shredded wheat/bran cereals | Wang 2020  Zhang 2020 | Gastric |
| Hansson et al. 1993 [40] | Case-control (Sweden)  338 cases (218 men, 120 women), 679 controls (444 men, 235 women) | FFQ | Whole meal bread | Zhang 2020  Jacobs 1998 | Gastric |
| Wu-Williams et al. 1990 [41] | Case-control (United States)  137 cases, 137 controls  (men only) | FFQ | Whole grain bread | Zhang 2020  Jacobs 1998 | Gastric |
| Chatenoud et al. 1999 [42] | Case-control (Italy)  745 cases (456 men, 289 women), 3,526 controls (2,069 men, 1,457 women) | FFQ | Not defined | Xu 2019  Zhang 2020 | Gastric |
| Lissowska et al. 2004 [43] | Case-control (Poland)  274 cases (175 men, 99 women), 463 controls (304 men, 159 women) | FFQ | Cooked groats, hot cooked cereal soup, dark bread | Xu 2019  Zhang 2020 | Gastric |
| Mathew et al. 2000 [44] | Case-control (India)  194 cases (151 men, 43 women), 305 controls (228 men, 77 women) | FFQ | Not defined | Wang 2020 | Gastric |
| Jedrychowski et al. 1992 [45] | Case-control (Poland)  741 cases, 741 controls  (sex not indicated) | Structured Questionnaire | Whole meal bread | Zhang 2020 | Gastric |
| Munoz et al. 1997 [46] | Case-control (Italy)  722 cases, 2024 controls  (sex not indicated) | FFQ | Not defined | Zhang 2020 | Gastric |
| Tuyns et al. 1992 [47] | Case-control (Belgium)  449 cases (249 men, 200 women); 3,524 controls (1,786 men, 1,738 women) | FFQ | Whole meal bread | Jacobs 1998 | Gastric |
| Trichopoulos et al. 1985 [48] | Case-control (Greece)  110 cases (57 men, 53 women); 100 controls (49 men, 51 women) | FFQ | Brown bread | Jacobs 1998 | Gastric |
| Chan et al. 2007 [49] | Case-control (United States)  532 cases (291 men, 241 women), 1,701 controls (883 men, 818 women) | FFQ | Brown rice, tortillas, popcorn, other grains  (bulgar, couscous, kasha, etc.), oat bran added to food, other  bran added to food, wheat germ, and cooked oatmeal/  oat bran | Lei 2016 | Pancreatic |
| Jansen et al. 2011 [50] | Case-control (United States)  384 cases (221 men, 163 women), 983 controls (483 men, 500 women) | FFQ | Not defined | Lei 2016 | Pancreatic |
| La Vecchia et al. 2003 [51] | Case-control (Italy)  402 cases (258 men, 144 women), 10,058 controls | FFQ | Whole grain bread or pasta | Lei 2016 | Pancreatic |
| Stolzenberg-Soloman et al. 2002 [52] | Alpha-Tocopherol, Beta-Carotene Cancer Prevention Study (Finland)  27,111 men (smokers) | FFQ | Wheat products | Lei 2016 | Pancreatic |
| Gold et al. 1985 [53] | Case-control (United States)  201 cases (47% men); 201 controls (47% men) | FFQ | Whole grain breads | Jacobs 1998 | Pancreatic |
| Olsen et al. 1989 [54] | Case-control (United States)  212 cases; 220 controls (men only) | FFQ | Whole wheat bread | Jacobs 1998 | Pancreatic |
| Mack et al. 1986 [55] | Case-control (United States)  490 cases (282 men, 208 women); 490 controls (282 men, 208 women) | FFQ | Whole grain bread | Jacobs 1998 | Pancreatic |
| Bueno de Mesquita et al. 1991 [56] | Case-control (Netherlands)  164 cases (90 men, 74 women); 480 controls (232 men, 248 women) | FFQ | Whole meal bread | Jacobs 1998 | Pancreatic |
| Egeberg et al. 2011 [57] | Diet, Cancer and Health Study (Denmark)  26,691 men | FFQ | Whole grain products from breads and cereals, including whole grain bread and whole grain rye bread, and oatmeal | Wang 2015  Reynolds 2019  Zhang 2018 | Prostate |
| Nimptisch et al. 2011 [58] | Health Professionals Follow-up Study (United States)  49,934 men | FFQ | Whole wheat and whole wheat flour, whole oats and whole oat flour, whole cornmeal and whole corn flour, whole rye and whole rye flour, whole barley, bulgur, buckwheat, brown rice and brown rice  flour, popcorn, amaranth, psyllium | Wang 2015  Reynolds 2019  Zhang 2018 | Prostate |
| Drake et al. 2012 [59] | Malmo Diet and Cancer Study (Sweden)  8,128 men | FFQ | Not defined | Wang 2015  Reynolds 2019 | Prostate |
| Tabung et al. 2012 [60] | Case-only study (North Carolina-Louisiana Prostate Cancer Project, United States)  1,923 men | National Cancer Institute diet history questionnaire | Not defined | Zhang | Prostate |
| Jain et al. 1999 [61] | Case-control (Canada)  617 cases, 636 controls | FFQ | Whole grain bread and breakfast cereals | Wang 2015 | Prostate |
| Deneo-Pellegrini et al. 1999 [62] | Case-control (Uruguay)  175 cases, 233 controls | FFQ | Not defined | Wang 2015 | Prostate |
| Lewis et al. 2009 [63] | Case-control (United States)  382 cases, 478 controls | FFQ | Not defined | Wang 2015 | Prostate |
| Hardin et al. 2011 [64] | Case-control (United States) 470 cases, 512 controls | FFQ | Dark breads, including dark bagels and rolls; cooked cereals and grits | Wang 2015 | Prostate |
| Chatenoud et al. 1998 [65] | Case-control (Italy)  127 cases, 3,332 controls | FFQ | Whole grain bread or pasta | Wang 2015 | Prostate |
| La Vecchia et al. 1987 [66] | Case-control (Italy)  2,389 women (1,108 cases, 1,281 controls) | FFQ | Whole grain bread or pasta | Jacobs 1998  Xiao 2018 | Breast |
| Levi et al. 1993 [67] | Case-control (Switzerland)  425 women (107 cases, 318 controls) | FFQ | Whole grain bread and pasta | Jacobs 1998  Xiao 2018 | Breast |
| Egeberg et al. 2009 [68] | Diet, Cancer and Health Study (Denmark)  25,278 women | FFQ | Whole grain products from breads and cereals, including whole grain bread and whole grain rye bread, and oatmeal | Xiao 2018 | Breast |
| Farvid et al. 2016 [69] | Nurses’ Health Study II (United States)  90,516 women | FFQ | Dark bread, cooked oatmeal/oat bran, other cooked breakfast cereal, brown rice, other grains, corn tortillas, added bran and germ | Xiao 2018 | Breast |
| Sonestedt et al. 2008 [70] | Malmo Diet and Cancer Study (Sweden)  15,773 women | FFQ and 7-d modified diet history | Not defined | Xiao 2018 | Breast |
| Nicodemis et al. 2001 [71] | Iowa Women’s Health Study (United States)  29,119 women | FFQ | Breakfast cereals with a 25% or greater amount of whole  grain or bran by weight, or in which the ingredient list  included whole grain and the cereal contained at least  2 g of fiber per serving, were considered whole grain cereals; dark bread, brown rice | Xiao 2018 | Breast |
| Adzersen et al. 2003 [72] | Case-control (Germany)  663 women (310 cases, 363 controls) | FFQ | Whole grain bread  and rice, rolled oats, muesli, and cornflakes | Xiao 2018 | Breast |
| Chatenoud et al. 1998 [65] | Case-control (Italy)  8,142 women (3,412 cases, 4,770 controls) | FFQ | Whole grain bread or pasta | Xiao 2018 | Breast |
| Mourouti et al. 2016 [73] | Case-control (Greece)  500 women (250 cases, 250 controls) | FFQ | Whole grain foods (including whole  grain bread, whole grain cereals,  oatmeal, whole wheat pasta,  brown or wild rice) | Xiao 2018 | Breast |
| Tajaddini et al. 2015 [74] | Case-control (Iran)  615 women (306 cases, 309 controls) | FFQ | Whole wheat bread (Sangak, Taftoon,  Barbari, barley, corn flakes and sprouts) | Xiao 2018 | Breast |
| Yun et al. 2010 [75] | Case-control (South Korea)  724 women (362 cases, 362 controls) | FFQ | Mixed brown rice | Xiao 2018 | Breast |
| Levi et al. 1993 [76] | Case-control (Switzerland and Italy)  274 cases, 572 controls | FFQ | Whole grain bread and pasta | Jacobs 1998 | Endometrial |
| La Vecchia et al. 1986 [77] | Case-control (Italy)  206 cases, 206 controls | FFQ | Whole-grain bread or pasta | Jacobs 1998 | Endometrial |
| Goodman et al. 1997 [78] | Case-control (Hawaii, United States)  332 cases, 511 controls | FFQ | Whole grain and mixed grain breads, dark breads, bran muffins, brown rice;  Whole grain cereals, including cooked cereals such as oat bran, Wheatena, or seven grain, cold high fiber cereals and fortified cereals such as bran, natural grain, Muesli, Cheerios, Wheaties, Nutri Grain, and Grapenuts | Jacobs 1998 | Endometrial |
| Kasum et al. 2002 [79] | Iowa Women’s Health Study (United States)  34,651 women | FFQ | Foods containing >25% whole grains and/or bran by weight | Xu 2019  Zhang 2020 | Esophageal  Gastric |
| Levi et al. 2000 [80] | Case-control (Switzerland)  101 cases (82 men, 19 women); 349 controls (302 men, 47 women) | FFQ | Whole wheat bread and cereals | Zhang 2020 | Esophageal |
| Chen et al. 2002 [81] | Case-control (United States)  124 cases (109 men, 15 women); 449 controls (258 men, 191 women) | FFQ | Dark bread | Zhang 2020 | Esophageal |
| Jessri et al. 2012 [82] | Case-control (Iran)  47 cases; 96 controls  (sex not indicated) | FFQ | Whole grain cereals | Zhang 2020 | Esophageal |
| Sewram et al. 2014 [83] | Case-control (South Africa)  670 cases (334 men, 336 women); 1,188 controls (621 men, 567 women) | FFQ | Wheat-based products (whole grain not defined) | Zhang 2020 | Esophageal |
| Skeie et al. 2016 [84] | HELGA Cohort (Sweden) 38,530 men  75,463 women | FFQ | Whole grain bread, or >75% of the products included in the whole grain category contained some whole grains (crisp bread and breakfast cereals) | Zhang 2020 | Esophageal |
| Yu et al. 1988 [85] | Case-control (United States)  275 cases (187 men, 88 women); 275 controls (187 men, 88 women) | FFQ | Whole grain bread | Jacobs 1998 | Esophageal |
| Decarli et al. 1987 [86] | Case-control (Italy)  105 cases (83 men, 22 women); 348 controls (236 men, 112 women) | FFQ | Whole meal bread or pasta | Jacobs 1998 | Esophageal |
| Franceschi et al. 1992 [87] | Case-control (Italy)  206 cases, 726 controls (men only) | FFQ | Whole grain bread and pasta | Jacobs 1998 | Oral |
| McLaughlin et al. 1988 [88] | Case-control (United State)  871 cases (67% men), 979 controls (67%) | FFQ | Whole wheat breads | Jacobs 1998 | Oral |
| Winn et al. 1984 [89] | Case-control (United State)  227 cases, 405 controls (women only) | FFQ | Whole grain breads and cereals | Jacobs 1998 | Oral |
| La Vecchia et al. 1990 [90] | Case-control (Italy)  110 cases, 843 controls (men only) | FFQ | Whole meal bread or pasta | Jacobs 1998 | Oral |
| Giles et al. 1994 [91] | Case-control (Australia)  409 cases (243 men, 166 women), 409 controls (243 men, 166 women) | FFQ | Whole grain bread, whole grain cereal, and whole grain pasta | Jacobs 1998 | Brain |
| Boeing et al. 1993 [92] | Case-control (Germany)  115 cases; 418 controls (men and women included, specific numbers not provided) | FFQ | Brown or whole meal bread | Jacobs 1998 | Brain |
| Tavani et al. 1997 [93] | Case-control (Italy) 429 cases (249 men, 180 women); 1,157 controls (709 men, 448 women) | FFQ | Not defined | Jacobs 1998 | Non-Hodgkin’s Lymphoma |
| Franceschi et al. 1989 [94] | Case-control (Italy)  208 case (110 men, 98 women); 401 controls (215 men, 186 women) | FFQ | Whole grain bread and pasta | Jacobs 1998 | Non-Hodgkin’s Lymphoma |

FFQ: Food Frequency Questionnaire

**References**

1. Huang, T.; Xu, M.; Lee, A.; Cho, S.; Qi, L. Consumption of whole grains and cereal fiber and total and cause-specific mortality: prospective analysis of 367,442 individuals. *BMC Med* **2015**, *13*, 59, doi:10.1186/s12916-015-0294-7.

2. Wu, H.; Flint, A.J.; Qi, Q.; van Dam, R.M.; Sampson, L.A.; Rimm, E.B.; Holmes, M.D.; Willett, W.C.; Hu, F.B.; Sun, Q. Association between dietary whole grain intake and risk of mortality: two large prospective studies in US men and women. *JAMA Intern Med* **2015**, *175*, 373-384, doi:10.1001/jamainternmed.2014.6283.

3. Jacobs, D.R., Jr.; Andersen, L.F.; Blomhoff, R. Whole-grain consumption is associated with a reduced risk of noncardiovascular, noncancer death attributed to inflammatory diseases in the Iowa Women's Health Study. *Am J Clin Nutr* **2007**, *85*, 1606-1614, doi:10.1093/ajcn/85.6.1606.

4. Johnsen, N.F.; Frederiksen, K.; Christensen, J.; Skeie, G.; Lund, E.; Landberg, R.; Johansson, I.; Nilsson, L.M.; Halkjaer, J.; Olsen, A., et al. Whole-grain products and whole-grain types are associated with lower all-cause and cause-specific mortality in the Scandinavian HELGA cohort. *Br J Nutr* **2015**, *114*, 608-623, doi:10.1017/S0007114515001701.

5. Buil-Cosiales, P.; Zazpe, I.; Toledo, E.; Corella, D.; Salas-Salvado, J.; Diez-Espino, J.; Ros, E.; Fernandez-Creuet Navajas, J.; Santos-Lozano, J.M.; Aros, F., et al. Fiber intake and all-cause mortality in the Prevencion con Dieta Mediterranea (PREDIMED) study. *Am J Clin Nutr* **2014**, *100*, 1498-1507, doi:10.3945/ajcn.114.093757.

6. Jacobs, D.R., Jr.; Meyer, H.E.; Solvoll, K. Reduced mortality among whole grain bread eaters in men and women in the Norwegian County Study. *Eur J Clin Nutr* **2001**, *55*, 137-143, doi:10.1038/sj.ejcn.1601133.

7. Key, T.J.; Thorogood, M.; Appleby, P.N.; Burr, M.L. Dietary habits and mortality in 11,000 vegetarians and health conscious people: results of a 17 year follow up. *BMJ* **1996**, *313*, 775-779, doi:10.1136/bmj.313.7060.775.

8. Appleby, P.N.; Key, T.J.; Burr, M.L.; Thorogood, M. Mortality and fresh fruit consumption. *IARC Sci Publ* **2002**, *156*, 131-133.

9. Aarestrup, J.; Kyro, C.; Christensen, J.; Kristensen, M.; Wurtz, A.M.; Johnsen, N.F.; Overvad, K.; Tjonneland, A.; Olsen, A. Whole grain, dietary fiber, and incidence of endometrial cancer in a Danish cohort study. *Nutr Cancer* **2012**, *64*, 1160-1168, doi:10.1080/01635581.2012.723786.

10. Jacobs, D.R., Jr.; Meyer, K.A.; Kushi, L.H.; Folsom, A.R. Is whole grain intake associated with reduced total and cause-specific death rates in older women? The Iowa Women's Health Study. *Am J Public Health* **1999**, *89*, 322-329, doi:10.2105/ajph.89.3.322.

11. Larsson, S.C.; Giovannucci, E.; Bergkvist, L.; Wolk, A. Whole grain consumption and risk of colorectal cancer: a population-based cohort of 60,000 women. *Br J Cancer* **2005**, *92*, 1803-1807, doi:10.1038/sj.bjc.6602543.

12. Schatzkin, A.; Mouw, T.; Park, Y.; Subar, A.F.; Kipnis, V.; Hollenbeck, A.; Leitzmann, M.F.; Thompson, F.E. Dietary fiber and whole-grain consumption in relation to colorectal cancer in the NIH-AARP Diet and Health Study. *Am J Clin Nutr* **2007**, *85*, 1353-1360, doi:10.1093/ajcn/85.5.1353.

13. Kyro, C.; Skeie, G.; Loft, S.; Landberg, R.; Christensen, J.; Lund, E.; Nilsson, L.M.; Palmqvist, R.; Tjonneland, A.; Olsen, A. Intake of whole grains from different cereal and food sources and incidence of colorectal cancer in the Scandinavian HELGA cohort. *Cancer Causes Control* **2013**, *24*, 1363-1374, doi:10.1007/s10552-013-0215-z.

14. Egeberg, R.; Olsen, A.; Loft, S.; Christensen, J.; Johnsen, N.F.; Overvad, K.; Tjonneland, A. Intake of wholegrain products and risk of colorectal cancers in the Diet, Cancer and Health cohort study. *Br J Cancer* **2010**, *103*, 730-734, doi:10.1038/sj.bjc.6605806.

15. Bakken, T.; Braaten, T.; Olsen, A.; Kyro, C.; Lund, E.; Skeie, G. Consumption of Whole-Grain Bread and Risk of Colorectal Cancer among Norwegian Women (the NOWAC Study). *Nutrients* **2016**, *8*, doi:10.3390/nu8010040.

16. Fung, T.T.; Hu, F.B.; Wu, K.; Chiuve, S.E.; Fuchs, C.S.; Giovannucci, E. The Mediterranean and Dietary Approaches to Stop Hypertension (DASH) diets and colorectal cancer. *Am J Clin Nutr* **2010**, *92*, 1429-1435, doi:10.3945/ajcn.2010.29242.

17. McCarl, M.; Harnack, L.; Limburg, P.J.; Anderson, K.E.; Folsom, A.R. Incidence of colorectal cancer in relation to glycemic index and load in a cohort of women. *Cancer Epidemiol Biomarkers Prev* **2006**, *15*, 892-896, doi:10.1158/1055-9965.EPI-05-0700.

18. Pietinen, P.; Malila, N.; Virtanen, M.; Hartman, T.J.; Tangrea, J.A.; Albanes, D.; Virtamo, J. Diet and risk of colorectal cancer in a cohort of Finnish men. *Cancer Causes Control* **1999**, *10*, 387-396, doi:10.1023/a:1008962219408.

19. Peters, R.K.; Garabrant, D.H.; Yu, M.C.; Mack, T.M. A case-control study of occupational and dietary factors in colorectal cancer in young men by subsite. *Cancer Res* **1989**, *49*, 5459-5468.

20. Centonze, S.; Boeing, H.; Leoci, C.; Guerra, V.; Misciagna, G. Dietary habits and colorectal cancer in a low-risk area. Results from a population-based case-control study in southern Italy. *Nutr Cancer* **1994**, *21*, 233-246, doi:10.1080/01635589409514322.

21. Slattery, M.L.; Berry, T.D.; Potter, J.; Caan, B. Diet diversity, diet composition, and risk of colon cancer (United States). *Cancer Causes Control* **1997**, *8*, 872-882, doi:10.1023/a:1018416412906.

22. Sanjoaquin, M.A.; Appleby, P.N.; Thorogood, M.; Mann, J.I.; Key, T.J. Nutrition, lifestyle and colorectal cancer incidence: a prospective investigation of 10998 vegetarians and non-vegetarians in the United Kingdom. *Br J Cancer* **2004**, *90*, 118-121, doi:10.1038/sj.bjc.6601441.

23. Vulcan, A.; Brandstedt, J.; Manjer, J.; Jirstrom, K.; Ohlsson, B.; Ericson, U. Fibre intake and incident colorectal cancer depending on fibre source, sex, tumour location and Tumour, Node, Metastasis stage. *Br J Nutr* **2015**, *114*, 959-969, doi:10.1017/S0007114515002743.

24. Angelo, S.N.; Lourenco, G.J.; Magro, D.O.; Nascimento, H.; Oliveira, R.A.; Leal, R.F.; Ayrizono Mde, L.; Fagundes, J.J.; Coy, C.S.; Lima, C.S. Dietary risk factors for colorectal cancer in Brazil: a case control study. *Nutr J* **2016**, *15*, 20, doi:10.1186/s12937-016-0139-z.

25. Williams, C.D.; Satia, J.A.; Adair, L.S.; Stevens, J.; Galanko, J.; Keku, T.O.; Sandler, R.S. Dietary patterns, food groups, and rectal cancer risk in Whites and African-Americans. *Cancer Epidemiol Biomarkers Prev* **2009**, *18*, 1552-1561, doi:10.1158/1055-9965.EPI-08-1146.

26. Abu Mweis, S.S.; Tayyem, R.F.; Shehadah, I.; Bawadi, H.A.; Agraib, L.M.; Bani-Hani, K.E.; Al-Jaberi, T.; Al-Nusairr, M. Food groups and the risk of colorectal cancer: results from a Jordanian case-control study. *Eur J Cancer Prev* **2015**, *24*, 313-320, doi:10.1097/CEJ.0000000000000089.

27. Tayyem, R.F.; Bawadi, H.A.; Shehadah, I.; Agraib, L.M.; Al-Awwad, N.J.; Heath, D.D.; Bani-Hani, K.E. Consumption of Whole Grains, Refined Cereals, and Legumes and Its Association With Colorectal Cancer Among Jordanians. *Integr Cancer Ther* **2016**, *15*, 318-325, doi:10.1177/1534735415620010.

28. He, X.; Wu, K.; Zhang, X.; Nishihara, R.; Cao, Y.; Fuchs, C.S.; Giovannucci, E.L.; Ogino, S.; Chan, A.T.; Song, M. Dietary intake of fiber, whole grains and risk of colorectal cancer: An updated analysis according to food sources, tumor location and molecular subtypes in two large US cohorts. *Int J Cancer* **2019**, *145*, 3040-3051, doi:10.1002/ijc.32382.

29. Um, C.Y.; Campbell, P.T.; Carter, B.; Wang, Y.; Gapstur, S.M.; McCullough, M.L. Association between grains, gluten and the risk of colorectal cancer in the Cancer Prevention Study-II Nutrition Cohort. *Eur J Nutr* **2020**, *59*, 1739-1749, doi:10.1007/s00394-019-02032-2.

30. Bidoli, E.; Franceschi, S.; Talamini, R.; Barra, S.; La Vecchia, C. Food consumption and cancer of the colon and rectum in north-eastern Italy. *Int J Cancer* **1992**, *50*, 223-229, doi:10.1002/ijc.2910500211.

31. Tuyns, A.J.; Kaaks, R.; Haelterman, M. Colorectal cancer and the consumption of foods: a case-control study in Belgium. *Nutr Cancer* **1988**, *11*, 189-204, doi:10.1080/01635588809513986.

32. La Vecchia, C.; Negri, E.; Decarli, A.; D'Avanzo, B.; Gallotti, L.; al., E. A case-control study of diet and colorectal cancer in Northern Italy. *Int J Cancer* **1988**, *41*, 492-498.

33. Pickle, L.W.; Greene, M.H.; Ziegler, R.G.; Toledo, A.; Hoover, R.; Lynch, H.T.; Fraumeni, J.F., Jr. Colorectal cancer in rural Nebraska. *Cancer Res* **1984**, *44*, 363-369.

34. McCullough, M.L.; Robertson, A.S.; Chao, A.; Jacobs, E.J.; Stampfer, M.J.; Jacobs, D.R.; Diver, W.R.; Calle, E.E.; Thun, M.J. A prospective study of whole grains, fruits, vegetables and colon cancer risk. *Cancer Causes Control* **2003**, *14*, 959-970, doi:10.1023/b:caco.0000007983.16045.a1.

35. Wu, K.; Hu, F.B.; Fuchs, C.; Rimm, E.B.; Willett, W.C.; Giovannucci, E. Dietary patterns and risk of colon cancer and adenoma in a cohort of men (United States). *Cancer Causes Control* **2004**, *15*, 853-862, doi:10.1007/s10552-004-1809-2.

36. La Vecchia, C.; Decarli, A.; Negri, E.; Parazzini, F. Epidemiological aspects of diet and cancer: a summary review of case-control studies from northern Italy. *Oncology* **1988**, *45*, 364-370, doi:10.1159/000226642.

37. Boeing, H.; Frentzel-Beyme, R.; Berger, M.; Berndt, V.; Gores, W.; Korner, M.; Lohmeier, R.; Menarcher, A.; Mannl, H.F.; Meinhardt, M., et al. Case-control study on stomach cancer in Germany. *Int J Cancer* **1991**, *47*, 858-864.

38. Boeing, H.; Jedrychowski, W.; Wahrendorf, J.; Popiela, T.; Tobiasz-Adamczyk, B.; Kulig, A. Dietary risk factors in intestinal and diffuse types of stomach cancer: a multicenter case-control study in Poland. *Cancer Causes Control* **1991**, *2*, 227-233, doi:10.1007/BF00052138.

39. McCullough, M.L.; Robertson, A.S.; Jacobs, E.J.; Chao, A.; Calle, E.E.; Thun, M.J. A prospective study of diet and stomach cancer mortality in United States men and women. *Cancer Epidemiol Biomarkers Prev* **2001**, *10*, 1201-1205.

40. Hansson, L.E.; Nyren, O.; Bergstrom, R.; Wolk, A.; Lindgren, A.; Baron, J.; Adami, H.O. Diet and risk of gastric cancer. A population-based case-control study in Sweden. *Int J Cancer* **1993**, *55*, 181-189.

41. Wu-Williams, A.H.; Yu, M.C.; Mack, T.M. Life-style, workplace, and stomach cancer by subsite in young men of Los Angeles County. *Cancer Res* **1990**, *50*, 2569-2576.

42. Chatenoud, L.; La Vecchia, C.; Franceschi, S.; Tavani, A.; Jacobs, D.R., Jr.; Parpinel, M.T.; Soler, M.; Negri, E. Refined-cereal intake and risk of selected cancers in italy. *Am J Clin Nutr* **1999**, *70*, 1107-1110, doi:10.1093/ajcn/70.6.1107.

43. Lissowska, J.; Gail, M.H.; Pee, D.; Groves, F.D.; Sobin, L.H.; Nasierowska-Guttmejer, A.; Sygnowska, E.; Zatonski, W.; Blot, W.J.; Chow, W.H. Diet and stomach cancer risk in Warsaw, Poland. *Nutr Cancer* **2004**, *48*, 149-159, doi:10.1207/s15327914nc4802_4.

44. Mathew, A.; Gangadharan, P.; Varghese, C.; Nair, M.K. Diet and stomach cancer: a case-control study in South India. *Eur J Cancer Prev* **2000**, *9*, 89-97, doi:10.1097/00008469-200004000-00004.

45. Jedrychowski, W.; Boeing, H.; Popiela, T.; Wahrendorf, J.; Tobiasz-Adamczyk, B.; Kulig, J. Dietary practices in households as risk factors for stomach cancer: a familial study in Poland. *Eur J Cancer Prev* **1992**, *1*, 297-304, doi:10.1097/00008469-199206000-00004.

46. Munoz, S.E.; Ferraroni, M.; La Vecchia, C.; Decarli, A. Gastric cancer risk factors in subjects with family history. *Cancer Epidemiol Biomarkers Prev* **1997**, *6*, 137-140.

47. Tuyns, A.J.; Kaaks, R.; Haelterman, M.; Riboli, E. Diet and gastric cancer. A case-control study in Belgium. *Int J Cancer* **1992**, *51*, 1-6, doi:10.1002/ijc.2910510102.

48. Trichopoulos, D.; Ouranos, G.; Day, N.E.; Tzonou, A.; Manousos, O.; Papadimitriou, C.; Trichopoulos, A. Diet and cancer of the stomach: a case-control study in Greece. *Int J Cancer* **1985**, *36*, 291-297.

49. Chan, J.M.; Wang, F.; Holly, E.A. Whole grains and risk of pancreatic cancer in a large population-based case-control study in the San Francisco Bay Area, California. *Am J Epidemiol* **2007**, *166*, 1174-1185, doi:10.1093/aje/kwm194.

50. Jansen, R.J.; Robinson, D.P.; Stolzenberg-Solomon, R.Z.; Bamlet, W.R.; de Andrade, M.; Oberg, A.L.; Hammer, T.J.; Rabe, K.G.; Anderson, K.E.; Olson, J.E., et al. Fruit and vegetable consumption is inversely associated with having pancreatic cancer. *Cancer Causes Control* **2011**, *22*, 1613-1625, doi:10.1007/s10552-011-9838-0.

51. La Vecchia, C.; Chatenoud, L.; Negri, E.; Franceschi, S. Session: whole cereal grains, fibre and human cancer wholegrain cereals and cancer in Italy. *Proc Nutr Soc* **2003**, *62*, 45-49, doi:10.1079/PNS2002235.

52. Stolzenberg-Solomon, R.Z.; Pietinen, P.; Taylor, P.R.; Virtamo, J.; Albanes, D. Prospective study of diet and pancreatic cancer in male smokers. *Am J Epidemiol* **2002**, *155*, 783-792, doi:10.1093/aje/155.9.783.

53. Gold, E.B.; Gordis, L.; Diener, M.D.; Seltser, R.; Boitnott, J.K.; Bynum, T.E.; Hutcheon, D.F. Diet and other risk factors for cancer of the pancreas. *Cancer* **1985**, *55*, 460-467, doi:10.1002/1097-0142(19850115)55:2<460::aid-cncr2820550229>3.0.co;2-v.

54. Olsen, G.W.; Mandel, J.S.; Gibson, R.W.; Wattenberg, L.W.; Schuman, L.M. A case-control study of pancreatic cancer and cigarettes, alcohol, coffee and diet. *Am J Public Health* **1989**, *79*, 1016-1019, doi:10.2105/ajph.79.8.1016.

55. Mack, T.M.; Yu, M.C.; Hanisch, R.; Henderson, B.E. Pancreas cancer and smoking, beverage consumption, and past medical history. *J Natl Cancer Inst* **1986**, *76*, 49-60.

56. Bueno de Mesquita, H.B.; Maisonneuve, P.; Runia, S.; Moerman, C.J. Intake of foods and nutrients and cancer of the exocrine pancreas: a population-based case-control study in The Netherlands. *Int J Cancer* **1991**, *48*, 540-549, doi:10.1002/ijc.2910480411.

57. Egeberg, R.; Olsen, A.; Christensen, J.; Johnsen, N.F.; Loft, S.; Overvad, K.; Tjonneland, A. Intake of whole-grain products and risk of prostate cancer among men in the Danish Diet, Cancer and Health cohort study. *Cancer Causes Control* **2011**, *22*, 1133-1139, doi:10.1007/s10552-011-9789-5.

58. Nimptsch, K.; Kenfield, S.; Jensen, M.K.; Stampfer, M.J.; Franz, M.; Sampson, L.; Brand-Miller, J.C.; Willett, W.C.; Giovannucci, E. Dietary glycemic index, glycemic load, insulin index, fiber and whole-grain intake in relation to risk of prostate cancer. *Cancer Causes Control* **2011**, *22*, 51-61, doi:10.1007/s10552-010-9671-x.

59. Drake, I.; Sonestedt, E.; Gullberg, B.; Ahlgren, G.; Bjartell, A.; Wallstrom, P.; Wirfalt, E. Dietary intakes of carbohydrates in relation to prostate cancer risk: a prospective study in the Malmo Diet and Cancer cohort. *Am J Clin Nutr* **2012**, *96*, 1409-1418, doi:10.3945/ajcn.112.039438.

60. Tabung, F.; Steck, S.E.; Su, L.J.; Mohler, J.L.; Fontham, E.T.; Bensen, J.T.; Hebert, J.R.; Zhang, H.; Arab, L. Intake of grains and dietary fiber and prostate cancer aggressiveness by race. *Prostate Cancer* **2012**, *2012*, 323296, doi:10.1155/2012/323296.

61. Jain, M.G.; Hislop, G.T.; Howe, G.R.; Ghadirian, P. Plant foods, antioxidants, and prostate cancer risk: findings from case-control studies in Canada. *Nutr Cancer* **1999**, *34*, 173-184, doi:10.1207/S15327914NC3402_8.

62. Deneo-Pellegrini, H.; De Stefani, E.; Ronco, A.; Mendilaharsu, M. Foods, nutrients and prostate cancer: a case-control study in Uruguay. *Br J Cancer* **1999**, *80*, 591-597, doi:10.1038/sj.bjc.6690396.

63. Lewis, J.E.; Soler-Vila, H.; Clark, P.E.; Kresty, L.A.; Allen, G.O.; Hu, J.J. Intake of plant foods and associated nutrients in prostate cancer risk. *Nutr Cancer* **2009**, *61*, 216-224, doi:10.1080/01635580802419756.

64. Hardin, J.; Cheng, I.; Witte, J.S. Impact of consumption of vegetable, fruit, grain, and high glycemic index foods on aggressive prostate cancer risk. *Nutr Cancer* **2011**, *63*, 860-872, doi:10.1080/01635581.2011.582224.

65. Chatenoud, L.; Tavani, A.; La Vecchia, C.; Jacobs, D.R., Jr.; Negri, E.; Levi, F.; Franceschi, S. Whole grain food intake and cancer risk. *Int J Cancer* **1998**, *77*, 24-28, doi:10.1002/(sici)1097-0215(19980703)77:1<24::aid-ijc5>3.0.co;2-1.

66. La Vecchia, C.; Decarli, A.; Franceschi, S.; Gentile, A.; Negri, E.; Parazzini, F. Dietary factors and the risk of breast cancer. *Nutr Cancer* **1987**, *10*, 205-214, doi:10.1080/01635588709513958.

67. Levi, F.; La Vecchia, C.; Gulie, C.; Negri, E. Dietary factors and breast cancer risk in Vaud, Switzerland. *Nutr Cancer* **1993**, *19*, 327-335, doi:10.1080/01635589309514263.

68. Egeberg, R.; Olsen, A.; Loft, S.; Christensen, J.; Johnsen, N.F.; Overvad, K.; Tjonneland, A. Intake of whole grain products and risk of breast cancer by hormone receptor status and histology among postmenopausal women. *Int J Cancer* **2009**, *124*, 745-750, doi:10.1002/ijc.23992.

69. Farvid, M.S.; Cho, E.; Eliassen, A.H.; Chen, W.Y.; Willett, W.C. Lifetime grain consumption and breast cancer risk. *Breast Cancer Res Treat* **2016**, *159*, 335-345, doi:10.1007/s10549-016-3910-0.

70. Sonestedt, E.; Borgquist, S.; Ericson, U.; Gullberg, B.; Landberg, G.; Olsson, H.; Wirfalt, E. Plant foods and oestrogen receptor alpha- and beta-defined breast cancer: observations from the Malmo Diet and Cancer cohort. *Carcinogenesis* **2008**, *29*, 2203-2209, doi:10.1093/carcin/bgn196.

71. Nicodemus, K.K.; Jacobs, D.R., Jr.; Folsom, A.R. Whole and refined grain intake and risk of incident postmenopausal breast cancer (United States). *Cancer Causes Control* **2001**, *12*, 917-925.

72. Adzersen, K.H.; Jess, P.; Freivogel, K.W.; Gerhard, I.; Bastert, G. Raw and cooked vegetables, fruits, selected micronutrients, and breast cancer risk: a case-control study in Germany. *Nutr Cancer* **2003**, *46*, 131-137, doi:10.1207/S15327914NC4602_05.

73. Mourouti, N.; Kontogianni, M.D.; Papavagelis, C.; Psaltopoulou, T.; Kapetanstrataki, M.G.; Plytzanopoulou, P.; Vassilakou, T.; Malamos, N.; Linos, A.; Panagiotakos, D.B. Whole Grain Consumption and Breast Cancer: A Case-Control Study in Women. *J Am Coll Nutr* **2016**, *35*, 143-149, doi:10.1080/07315724.2014.963899.

74. Tajaddini, A.; Pourzand, A.; Sanaat, Z.; Pirouzpanah, S. Dietary resistant starch contained foods and breast cancer risk: a case-control study in northwest of Iran. *Asian Pac J Cancer Prev* **2015**, *16*, 4185-4192, doi:10.7314/apjcp.2015.16.10.4185.

75. Yun, S.H.; Kim, K.; Nam, S.J.; Kong, G.; Kim, M.K. The association of carbohydrate intake, glycemic load, glycemic index, and selected rice foods with breast cancer risk: a case-control study in South Korea. *Asia Pac J Clin Nutr* **2010**, *19*, 383-392.

76. Levi, F.; Franceschi, S.; Negri, E.; La Vecchia, C. Dietary factors and the risk of endometrial cancer. *Cancer* **1993**, *71*, 3575-3581, doi:10.1002/1097-0142(19930601)71:11<3575::aid-cncr2820711119>3.0.co;2-0.

77. La Vecchia, C.; Decarli, A.; Fasoli, M.; Gentile, A. Nutrition and diet in the etiology of endometrial cancer. *Cancer* **1986**, *57*, 1248-1253, doi:10.1002/1097-0142(19860315)57:6<1248::aid-cncr2820570631>3.0.co;2-v.

78. Goodman, M.T.; Wilkens, L.R.; Hankin, J.H.; Lyu, L.C.; Wu, A.H.; Kolonel, L.N. Association of soy and fiber consumption with the risk of endometrial cancer. *Am J Epidemiol* **1997**, *146*, 294-306, doi:10.1093/oxfordjournals.aje.a009270.

79. Kasum, C.M.; Jacobs, D.R., Jr.; Nicodemus, K.; Folsom, A.R. Dietary risk factors for upper aerodigestive tract cancers. *Int J Cancer* **2002**, *99*, 267-272, doi:10.1002/ijc.10341.

80. Levi, F.; Pasche, C.; Lucchini, F.; Chatenoud, L.; Jacobs, D.R., Jr.; La Vecchia, C. Refined and whole grain cereals and the risk of oral, oesophageal and laryngeal cancer. *Eur J Clin Nutr* **2000**, *54*, 487-489, doi:10.1038/sj.ejcn.1601043.

81. Chen, H.; Ward, M.H.; Graubard, B.I.; Heineman, E.F.; Markin, R.M.; Potischman, N.A.; Russell, R.M.; Weisenburger, D.D.; Tucker, K.L. Dietary patterns and adenocarcinoma of the esophagus and distal stomach. *Am J Clin Nutr* **2002**, *75*, 137-144, doi:10.1093/ajcn/75.1.137.

82. Jessri, M.; Rashidkhani, B.; Hajizadeh, B.; Jacques, P.F. Adherence to Mediterranean-style dietary pattern and risk of esophageal squamous cell carcinoma: a case-control study in Iran. *J Am Coll Nutr* **2012**, *31*, 338-351, doi:10.1080/07315724.2012.10720437.

83. Sewram, V.; Sitas, F.; O'Connell, D.; Myers, J. Diet and esophageal cancer risk in the Eastern Cape Province of South Africa. *Nutr Cancer* **2014**, *66*, 791-799, doi:10.1080/01635581.2014.916321.

84. Skeie, G.; Braaten, T.; Olsen, A.; Kyro, C.; Tjonneland, A.; Landberg, R.; Nilsson, L.M.; Wennberg, M.; Overvad, K.; Asli, L.A., et al. Intake of whole grains and incidence of oesophageal cancer in the HELGA Cohort. *Eur J Epidemiol* **2016**, *31*, 405-414, doi:10.1007/s10654-015-0057-y.

85. Yu, M.C.; Garabrant, D.H.; Peters, J.M.; Mack, T.M. Tobacco, alcohol, diet, occupation, and carcinoma of the esophagus. *Cancer Res* **1988**, *48*, 3843-3848.

86. Decarli, A.; Liati, P.; Negri, E.; Franceschi, S.; La Vecchia, C. Vitamin A and other dietary factors in the etiology of esophageal cancer. *Nutr Cancer* **1987**, *10*, 29-37, doi:10.1080/01635588709513938.

87. Franceschi, S.; Barra, S.; La Vecchia, C.; Bidoli, E.; Negri, E.; Talamini, R. Risk factors for cancer of the tongue and the mouth. A case-control study from northern Italy. *Cancer* **1992**, *70*, 2227-2233, doi:10.1002/1097-0142(19921101)70:9<2227::aid-cncr2820700902>3.0.co;2-z.

88. McLaughlin, J.K.; Gridley, G.; Block, G.; Winn, D.M.; Preston-Martin, S.; Schoenberg, J.B.; Greenberg, R.S.; Stemhagen, A.; Austin, D.F.; Ershow, A.G., et al. Dietary factors in oral and pharyngeal cancer. *J Natl Cancer Inst* **1988**, *80*, 1237-1243, doi:10.1093/jnci/80.15.1237.

89. Winn, D.M.; Ziegler, R.G.; Pickle, L.W.; Gridley, G.; Blot, W.J.; Hoover, R.N. Diet in the etiology of oral and pharyngeal cancer among women from the southern United States. *Cancer Res* **1984**, *44*, 1216-1222.

90. La Vecchia, C.; Negri, E.; D'Avanzo, B.; Franceschi, S.; Decarli, A.; Boyle, P. Dietary indicators of laryngeal cancer risk. *Cancer Res* **1990**, *50*, 4497-4500.

91. Giles, G.G.; McNeil, J.J.; Donnan, G.; Webley, C.; Staples, M.P.; Ireland, P.D.; Hurley, S.F.; Salzberg, M. Dietary factors and the risk of glioma in adults: results of a case-control study in Melbourne, Australia. *Int J Cancer* **1994**, *59*, 357-362, doi:10.1002/ijc.2910590311.

92. Boeing, H.; Schlehofer, B.; Blettner, M.; Wahrendorf, J. Dietary carcinogens and the risk for glioma and meningioma in Germany. *Int J Cancer* **1993**, *53*, 561-565, doi:10.1002/ijc.2910530406.

93. Tavani, A.; Pregnolato, A.; Negri, E.; Franceschi, S.; Serraino, D.; Carbone, A.; La Vecchia, C. Diet and risk of lymphoid neoplasms and soft tissue sarcomas. *Nutr Cancer* **1997**, *27*, 256-260, doi:10.1080/01635589709514535.

94. Franceschi, S.; Serraino, D.; Carbone, A.; Talamini, R.; La Vecchia, C. Dietary factors and non-Hodgkin's lymphoma: a case-control study in the northeastern part of Italy. *Nutr Cancer* **1989**, *12*, 333-341, doi:10.1080/01635588909514034.
