# Supplementary material for: Whole Grains, Refined Grains, and Cancer Risk: A Systematic Review of Meta-Analyses of Observational Studies
Source: Nutrients. 2020 Dec 7;12(12):3756. doi: 10.3390/nu12123756 (PMC7762239; doi:10.3390/nu12123756)
Supplement: Supplementary file 1 [file nutrients-12-03756-s001.zip › Supplementary Table 3.docx]

**Supplementary Table 3.** Characteristics of studies included in the meta-analyses on the association between refined grain intake and cancer risk

| **Study** | **Population** | **Dietary Assessment** | **Definition of refined grain** | **Meta-analysis included in** | **Cancer outcome or site** |
| --- | --- | --- | --- | --- | --- |
| Jacobs et al. 2007 [1] | Iowa Women’s Health Study (United States)  27,312 women | FFQ | Refined-grain breakfast cereal; English muffins, bagels, or rolls; pancakes or waffles; white rice; pasta  (e.g., spaghetti, noodles, etc.); pizza | Aune 2016 | Total cancer mortality |
| Wu et al. 2015 [2] | Nurses’ Health Study I 74,341 women  Health Professionals Follow-up Study  43,744 men  (both United States) | FFQ | Cold breakfast cereal white bread (while slice), white rice, pasta, English muffins/bagels/rolls, muffins or biscuits, pancakes or waffles, crackers, sweet rolls, pizza | Aune 2016 | Total cancer mortality |
| Larsson et al. 2005 [3] | Swedish Mammography Cohort (Sweden)  61,433 women | FFQ | Soft white bread, pasta, rice, pancakes or waffles, and sweet buns or biscuits | Schwingshackl 2018 | Colorectal  Colon |
| Sanjoaquin et al. 2004 [4] | Oxford Vegetarian Study (United Kingdom)  4,162 men  6,836 women | FFQ | White Bead | Schwingshackl 2018 | Colorectal |
| Wu et al. 2004 [5] | Health Professionals Follow-up Study (United States)  47,300 men | FFQ | Not defined | Aune 2011, Schwingshackl 2018 | Colon |
| Chatenoud et al. 1999 [6] | Case-control (Italy)  745 cases (456 men, 289 women); 3,526 controls (2,069 men, 1,457 women) | FFQ | Bread, pasta, rice | Xu 2018  Wang 2020 | Gastric |
| Lissowska et al. 2004 [7] | Case-control (Poland)  274 cases (175 men, 99 women), 463 controls (304 men, 159 women) | FFQ | Rice, spaghetti, other pasta, pizza, white rolls, French bread, white bread, sweetbread/rolls, cookies and doughnuts, other noodles, corn; includes sugar, layer cake, crisp biscuits-shortbread | Xu 2018  Wang 2020 | Gastric |
| Kasum et al. 2002 [8] | Iowa Women’s Health Study (United States)  34,651 women | FFQ | White bread including pita bread, cold breakfast cereals containing <25% whole grain or bran by weight,  English muffins, bagels or rolls, pancakes or waffles, white rice,  pasta and pizza. | Xu 2018 | Gastric |
| De Stefani et al. 2004 [9] | Case-control (Uruguay)  240 cases (168 men, 72 women), 960 controls (672 men, 288 women) | FFQ | Not defined  (white bread)* | Wang 2020 | Gastric |
| La Vecchia et al. 1987 [10] | Case-control (Italy)  206 cases (132 men, 74 women), 474 controls (321 men, 153 women) | FFQ | Not defined  (pasta or rice)* | Wang 2020 | Gastric |
| Mathew et al. 2000 [11] | Case-control (India)  194 cases (151 men, 43 women), 305 controls (228 men, 77 women) | FFQ | Not defined  (wheat)* | Wang 2020 | Gastric |
| Hansson et al. 1993 [12] | Case-control (Sweden)  338 cases (218 men, 120 women), 679 controls (444 men, 235 women) | FFQ | Not defined  (White bread)* | Wang 2020 | Gastric |
| Gao et al. 1999 [13] | Case-control (China)  153 cases (110 men, 43 women), 234 controls (154 men, 80 women) | FFQ | Not defined  (rice)* | Wang 2020 | Gastric |
| Hoshiyama et al. 1992 [14] | Case-control (Japan)  151 cases, 483 controls (men only) | FFQ | Not defined  (rice)* | Wang 2020 | Gastric |
| Inoue et al. 1996 [15] | Cohort Study (Japan)  2,552 men  5,373 women | FFQ | Not defined  (rice)* | Wang 2020 | Gastric |
| La Vecchia et al. 1988 [16] | Case-control (Italy)  206 cases (132 men, 74 women), 474 controls (321 men, 153 women) | FFQ | Not defined  (pasta or rice)* | Wang 2020 | Gastric |
| Li et al. 1989 [17] | Case-control (China)  1,244 cases (758 men, 486 women), 1,314 controls (789 men, 525 women) | FFQ | Not defined  (wheat)* | Wang 2020 | Gastric |
| Machida-Montani et al. 2004 [18] | Case-control (Japan)  122 cases (90 men, 32 women), 235 controls (159 men, 76 women) | FFQ | Not defined  (rice)* | Wang 2020 | Gastric |
| Navarro Silvera et al. 2008 [19] | Case-control (United States)  607 cases (461 men, 146 women), 687 controls (549 men, 138 women) | FFQ | Not defined  (refined grain)* | Wang 2020 | Gastric |
| Ramon et al. 1993 [20] | Case-control (Spain)  117 cases (99 men, 18 women), 234 controls (198 men, 36 women) | FFQ | Not defined  (rice)* | Wang 2020 | Gastric |
| Sumathi et al. 2009 [21] | Case-control (India)  89 cases (64 men, 25 women), 89 controls (64 men, 25 women) | FFQ | Not defined  (wheat)* | Wang 2020 | Gastric |
| You et al. 1988 [22] | Case-control (China)  564 cases (443 men, 121 women), 1,131 controls (888 men, 243 women) | FFQ | Not defined  (wheat)* | Wang 2020 | Gastric |

FFQ: Food Frequency Questionnaire. * Refined grain was not specifically defined in these studies used in the meta-analysis by Wang et al. The food in parentheses is the refined grain term appearing in the meta-analysis by Wang et al. and confirmed by this author’s review of each of the articles used by Wang et al.

**References**

1. Jacobs, D.R., Jr.; Andersen, L.F.; Blomhoff, R. Whole-grain consumption is associated with a reduced risk of noncardiovascular, noncancer death attributed to inflammatory diseases in the Iowa Women's Health Study. *Am J Clin Nutr* **2007**, *85*, 1606-1614, doi:10.1093/ajcn/85.6.1606.

2. Wu, H.; Flint, A.J.; Qi, Q.; van Dam, R.M.; Sampson, L.A.; Rimm, E.B.; Holmes, M.D.; Willett, W.C.; Hu, F.B.; Sun, Q. Association between dietary whole grain intake and risk of mortality: two large prospective studies in US men and women. *JAMA Intern Med* **2015**, *175*, 373-384, doi:10.1001/jamainternmed.2014.6283.

3. Larsson, S.C.; Giovannucci, E.; Bergkvist, L.; Wolk, A. Whole grain consumption and risk of colorectal cancer: a population-based cohort of 60,000 women. *Br J Cancer* **2005**, *92*, 1803-1807, doi:10.1038/sj.bjc.6602543.

4. Sanjoaquin, M.A.; Appleby, P.N.; Thorogood, M.; Mann, J.I.; Key, T.J. Nutrition, lifestyle and colorectal cancer incidence: a prospective investigation of 10998 vegetarians and non-vegetarians in the United Kingdom. *Br J Cancer* **2004**, *90*, 118-121, doi:10.1038/sj.bjc.6601441.

5. Wu, K.; Hu, F.B.; Fuchs, C.; Rimm, E.B.; Willett, W.C.; Giovannucci, E. Dietary patterns and risk of colon cancer and adenoma in a cohort of men (United States). *Cancer Causes Control* **2004**, *15*, 853-862, doi:10.1007/s10552-004-1809-2.

6. Chatenoud, L.; La Vecchia, C.; Franceschi, S.; Tavani, A.; Jacobs, D.R., Jr.; Parpinel, M.T.; Soler, M.; Negri, E. Refined-cereal intake and risk of selected cancers in italy. *Am J Clin Nutr* **1999**, *70*, 1107-1110, doi:10.1093/ajcn/70.6.1107.

7. Lissowska, J.; Gail, M.H.; Pee, D.; Groves, F.D.; Sobin, L.H.; Nasierowska-Guttmejer, A.; Sygnowska, E.; Zatonski, W.; Blot, W.J.; Chow, W.H. Diet and stomach cancer risk in Warsaw, Poland. *Nutr Cancer* **2004**, *48*, 149-159, doi:10.1207/s15327914nc4802_4.

8. Kasum, C.M.; Jacobs, D.R., Jr.; Nicodemus, K.; Folsom, A.R. Dietary risk factors for upper aerodigestive tract cancers. *Int J Cancer* **2002**, *99*, 267-272, doi:10.1002/ijc.10341.

9. De Stefani, E.; Correa, P.; Boffetta, P.; Deneo-Pellegrini, H.; Ronco, A.L.; Mendilaharsu, M. Dietary patterns and risk of gastric cancer: a case-control study in Uruguay. *Gastric Cancer* **2004**, *7*, 211-220, doi:10.1007/s10120-004-0295-2.

10. La Vecchia, C.; Negri, E.; Decarli, A.; D'Avanzo, B.; Franceschi, S. A case-control study of diet and gastric cancer in northern Italy. *Int J Cancer* **1987**, *40*, 484-489.

11. Mathew, A.; Gangadharan, P.; Varghese, C.; Nair, M.K. Diet and stomach cancer: a case-control study in South India. *Eur J Cancer Prev* **2000**, *9*, 89-97, doi:10.1097/00008469-200004000-00004.

12. Hansson, L.E.; Nyren, O.; Bergstrom, R.; Wolk, A.; Lindgren, A.; Baron, J.; Adami, H.O. Diet and risk of gastric cancer. A population-based case-control study in Sweden. *Int J Cancer* **1993**, *55*, 181-189, doi:10.1002/ijc.2910550203.

13. Gao, C.M.; Takezaki, T.; Ding, J.H.; Li, M.S.; Tajima, K. Protective effect of allium vegetables against both esophageal and stomach cancer: a simultaneous case-referent study of a high-epidemic area in Jiangsu Province, China. *Jpn J Cancer Res* **1999**, *90*, 614-621, doi:10.1111/j.1349-7006.1999.tb00791.x.

14. Hoshiyama, Y.; Sasaba, T. A case-control study of single and multiple stomach cancers in Saitama Prefecture, Japan. *Jpn J Cancer Res* **1992**, *83*, 937-943, doi:10.1111/j.1349-7006.1992.tb02004.x.

15. Inoue, M.; Tajima, K.; Kobayashi, S.; Suzuki, T.; Matsuura, A.; Nakamura, T.; Shirai, M.; Nakamura, S.; Inuzuka, K.; Tominaga, S. Protective factor against progression from atrophic gastritis to gastric cancer--data from a cohort study in Japan. *Int J Cancer* **1996**, *66*, 309-314, doi:10.1002/(SICI)1097-0215(19960503)66:3<309::AID-IJC7>3.0.CO;2-2.

16. La Vecchia, C.; Decarli, A.; Negri, E.; Parazzini, F. Epidemiological aspects of diet and cancer: a summary review of case-control studies from northern Italy. *Oncology* **1988**, *45*, 364-370, doi:10.1159/000226642.

17. Li, J.Y.; Ershow, A.G.; Chen, Z.J.; Wacholder, S.; Li, G.Y.; Guo, W.; Li, B.; Blot, W.J. A case-control study of cancer of the esophagus and gastric cardia in Linxian. *Int J Cancer* **1989**, *43*, 755-761, doi:10.1002/ijc.2910430502.

18. Machida-Montani, A.; Sasazuki, S.; Inoue, M.; Natsukawa, S.; Shaura, K.; Koizumi, Y.; Kasuga, Y.; Hanaoka, T.; Tsugane, S. Association of Helicobacter pylori infection and environmental factors in non-cardia gastric cancer in Japan. *Gastric Cancer* **2004**, *7*, 46-53, doi:10.1007/s10120-004-0268-5.

19. Navarro Silvera, S.A.; Mayne, S.T.; Risch, H.; Gammon, M.D.; Vaughan, T.L.; Chow, W.H.; Dubrow, R.; Schoenberg, J.B.; Stanford, J.L.; West, A.B., et al. Food group intake and risk of subtypes of esophageal and gastric cancer. *Int J Cancer* **2008**, *123*, 852-860, doi:10.1002/ijc.23544.

20. Ramon, J.M.; Serra, L.; Cerdo, C.; Oromi, J. Dietary factors and gastric cancer risk. A case-control study in Spain. *Cancer* **1993**, *71*, 1731-1735, doi:10.1002/1097-0142(19930301)71:5<1731::aid-cncr2820710505>3.0.co;2-x.

21. Sumathi, B.; Ramalingam, S.; Navaneethan, U.; Jayanthi, V. Risk factors for gastric cancer in South India. *Singapore Med J* **2009**, *50*, 147-151.

22. You, W.C.; Blot, W.J.; Chang, Y.S.; Ershow, A.G.; Yang, Z.T.; An, Q.; Henderson, B.; Xu, G.W.; Fraumeni, J.F., Jr.; Wang, T.G. Diet and high risk of stomach cancer in Shandong, China. *Cancer Res* **1988**, *48*, 3518-3523.
